# A trans-kingdom T6SS effector induces the fragmentation of the mitochondrial network and activates innate immune receptor NLRX1 to promote infection

**DOI:** 10.1038/s41467-023-36629-3

**Published:** 2023-02-16

**Authors:** Joana Sá-Pessoa, Sara López-Montesino, Kornelia Przybyszewska, Isabel Rodríguez-Escudero, Helina Marshall, Adelia Ova, Gunnar N. Schroeder, Peter Barabas, María Molina, Tim Curtis, Víctor J. Cid, José A. Bengoechea

**Affiliations:** 1 Wellcome-Wolfson Institute for Experimental Medicine, School of Medicine, Dentistry and Biomedical Sciences, Queen’s University Belfast, Belfast, UK; 2 Departamento de Microbiología y Parasitología, Facultad de Farmacia, Universidad Complutense de Madrid and Instituto Ramón y Cajal de Investigaciones Sanitarias, Madrid, Spain

**Keywords:** Cellular microbiology, Immune evasion, Bacterial secretion, Pathogens

## Abstract

Bacteria can inhibit the growth of other bacteria by injecting effectors using a type VI secretion system (T6SS). T6SS effectors can also be injected into eukaryotic cells to facilitate bacterial survival, often by targeting the cytoskeleton. Here, we show that the trans-kingdom antimicrobial T6SS effector VgrG4 from *Klebsiella pneumoniae* triggers the fragmentation of the mitochondrial network. VgrG4 colocalizes with the endoplasmic reticulum (ER) protein mitofusin 2. VgrG4 induces the transfer of Ca^2+^ from the ER to the mitochondria, activating Drp1 (a regulator of mitochondrial fission) thus leading to mitochondrial network fragmentation. Ca^2+^ elevation also induces the activation of the innate immunity receptor NLRX1 to produce reactive oxygen species (ROS). NLRX1-induced ROS limits NF-κB activation by modulating the degradation of the NF-κB inhibitor IκBα. The degradation of IκBα is triggered by the ubiquitin ligase SCF^β-TrCP^, which requires the modification of the cullin-1 subunit by NEDD8. VgrG4 abrogates the NEDDylation of cullin-1 by inactivation of Ubc12, the NEDD8-conjugating enzyme. Our work provides an example of T6SS manipulation of eukaryotic cells via alteration of the mitochondria.

## Abstract (web summary)

Bacteria can affect cellular processes in other bacteria and in eukaryotic cells by injecting effectors using a type VI secretion system (T6SS). Here, Sá-Pessoa et al. describe how a T6SS effector from the bacterial pathogen *Klebsiella pneumoniae* triggers the fragmentation of the mitochondrial network in eukaryotic cells.

## Introduction

Mitochondria originated from an endosymbiont α-*Proteobacterium* related to the genus *Rickettsia* more than 1.45 billion years ago^1^. Consistent with this bacterial origin, mitochondria are delimitated by a double membrane with an inner membrane characterized by the presence of cardiolipin and absence of cholesterol; they house a small circular genome referred as mitochondrial DNA (mtDNA), and a functional machinery for protein synthesis^2^. However, and in contrast to most bacteria, mitochondria are highly dynamic, and their morphology is balanced between two tightly regulated opposite events, fission and fusion. These processes are essential to maintain functional mitochondria when cells experience metabolic or environmental stresses^3^.

Mitochondria are fundamental to eukaryotic cell function. They are central for the energy production of the cell upon oxidation of tricarboxylic acid (TCA) cycle intermediates which involves the creation and harnessing of a membrane potential across the inner mitochondrial membrane^4^. Mitochondria also participate in the homeostasis of intracellular Ca^2+5^. High concentrations of Ca^2+^ can accumulate in the mitochondrial matrix due to the physical proximity to the endoplasmic reticulum (ER) and the presence of a regulated Ca^2+^ channel, the mitochondrial calcium uniporter (MCU) complex^5^. This Ca^2+^ entry may be associated with cell death via apoptosis^5^. Finally, mitochondria also contribute to cell intrinsic defence through ROS and by sensing infections^6^. Because of this central role in cellular processes, mitochondria are emerging as a prime target for pathogens^7^.

The bacterial origin of the mitochondria evokes an intriguing question: do pathogens interact with mitochondria employing the same systems they do deploy as defence against other bacterial competitors? For example, bacteria use the type VI secretion system (T6SS) to restrict the growth of other bacteria by delivering effectors into a target cell in a one-step process^8^. These antimicrobial T6SS effectors target essential elements of the bacterial cell such as the peptidoglycan, nucleic acids, or membrane phospholipids^8^. There are also a few examples of T6SS effectors delivered into mammalian cells^9^. These effectors mostly target the cytoskeleton and affect intracellular survival and bacterial internalization^9^. Yet surprisingly, no T6SS effectors targeting the mitochondria have been reported to date.

Recently, we have dissected the T6SS of the hypervirulent *Klebsiella pneumoniae* strain CIP52.145 (hereafter Kp52145). This strain encodes all the factors found in invasive *K. pneumoniae* strains^10,11^. We demonstrated the role of the T6SS in intra and inter bacterial species, and anti-fungal competition^12^. We characterized VgrG4 as new trans-kingdom T6SS effector intoxicating bacteria, yeast and fungi^12^. *vgrG4* is encoded in more than 10% of the *Klebsiella* genomes listed in NCBI (total 812), including isolates associated with human invasive infections and multidrug resistant strains. Truncation experiments demonstrated that the region of VgrG4 containing the domain DUF2345 is sufficient to exert microbial toxicity via ROS induction^12^. We termed this domain as ROS toxic domain (RTD)^12^ which is present in other VgrGs from *Escherichia coli*, *Acinetobacter baumannii*, and *Pseudomonas aeruginosa* strains.

The trans-kingdom nature of VgrG4 led us to investigate whether *K. pneumoniae* may exploit VgrG4 to control host cell biology. Here, we demonstrate that VgrG4 exerts a profound effect on mitochondrial dynamics in yeast and epithelial cells, resulting in fragmentation of the mitochondria network in lung cells. Strikingly, this fragmentation has no major effect on mitochondrial function. We demonstrate that VgrG4 induces the transfer of Ca^2+^ from the ER to the mitochondria to activate Drp1 to fragment the mitochondria. VgrG4-triggered transfer of Ca^2+^ also activates the mitochondria located innate immune receptor NLRX1. We show that VgrG4-dependent activation of NLRX1 limits the activation of inflammatory responses in a ROS-dependent manner in a process abrogating the post-translational modification of cullin-1 with NEDD8. Collectively, our findings identify a T6SS effector targeting the mitochondria and uncover a previously unknown signalling cascade exploited by a pathogen to evade innate responses.

## Results

### *K. pneumoniae* VgrG4 RTD domain induces oxidative stress and mitochondrial condensation in *Saccharomyces cerevisiae*

**Fig. 1:**
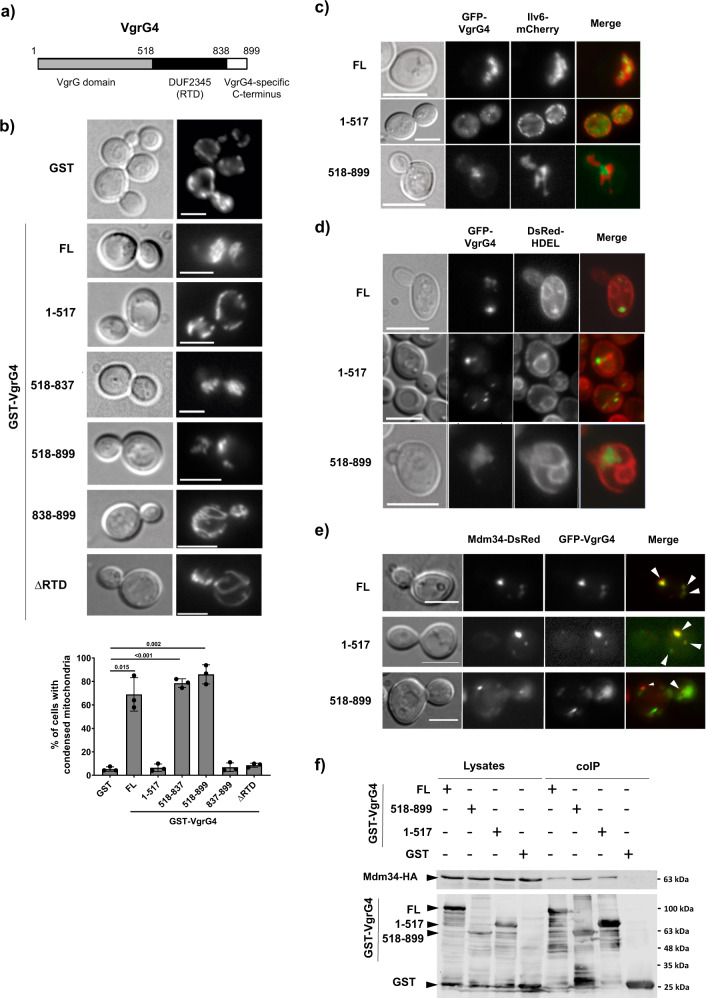
*K. pneumoniae* VgrG4 RTD domain causes mitochondrial condensation in *S. cerevisiae*, whereas its VgrG domain is sufficient to drive co-localization with the mitochondrial ERMES component Mdm34. **a** Scheme of the primary structure of VgrG4, depicting the domains considered in the development of truncated versions used in this work. **b** Yeast cells expressing the RTD domain of VgrG4 display condensed mitochondria. Cells were co-transformed with YEplac112-Ilv6-mCherry, a construct expressing a mCherry fusion to the mitochondrial Ilv6 protein, and the indicated GST-VgrG4 fusions or the empty pEG(KG) vector as a control (GST). Representative cells are shown and average percentages of cells with altered mitochondria with standard deviation error bars are shown in the graph; at least a total of 300 cells were analysed per smaple in three independent experiments. Two-tailed student’s *T*-test was applied for statistical significance between yeast cells harbouring the empty vector and the VgrG4 constructs with no adjustment for multiple comparison (*p* values indicated in the graph). **c** Fluorescence microscopy of co-expressed GFP-VgrG4 versions and the mitochondrial marker Ilv6-mCherry. Representative YPH499 strain transformants induced in galactose-containing media for 5 h are shown. **d** Co-localisation of GFP-VgrG4 spots and ER membranes. VHY87 cells, constitutively expressing the DsRed-HDEL marker, were transformed with pYES2-GFP-VgrG4 plasmids expressing the indicated versions, induced in galactose-containing media for 5 h and observed. **e** Bright field and fluorescence microscopy of the SLY001 strain co-transformed with the plasmids pAG424-Mdm34-DsRed (Mdm34 DsRed) and pYES2GFP-VgrG4 (FL) or pYES2GFP-VgrG4 1-517 (1-517) or pYES2GFP-VgrG4 518-899 (518-899). White arrows indicate co-localization events. Scales bars represent 5 µm in **c**, **d**, and **e**. **f** Immunoblots corresponding to a representative GST pull-down experiment on lysates from yeast cultures co-expressing Mdm34-HA. Images correspond to the same membrane hybridized with anti-HA (top panels) and anti-GST (lower panes) primary antibodies. Images are representative of three independent experiments.

We have demonstrated that VgrG4-induced antibacterial lethality is ROS-dependent being the 518-837 stretch of VgrG4 (RTD, see Fig. 1A) sufficient^12^. The fact that VgrG4 inhibits growth of the eukaryotic yeast model *S. cerevisiae* when endogenously expressed^12^ led us to assess whether VgrG4 expression was related to ROS production in yeast. Cells were stained with the ROS reporter dihydroethidium (DHE) and analysed by flow cytometry. Overexpression of GST-tagged full-length VgrG4 led to significant ROS production over control cells expressing GST alone in yeast cells. Among the truncated versions expressed, the one corresponding to the RTD domain alone led to higher ROS levels, whereas non-toxic versions, lacking the RTD, led to lower ROS levels (Supplementary Fig. 1A).

Oxidative stress is directly associated with mitochondria dysfunction in eukaryotic cells^13^, prompting us to investigate mitochondria morphology in yeast cells expressing different versions of VgrG4. To visualise yeast mitochondria in VgrG4-expressing cells, we used an Ilv6-mCherry fusion as a mitochondrial marker. GST-VgrG4 induced an aberrant mitochondrial morphology. Instead of a normal mitochondrial network extended along the cell, most cells displayed condensed mitochondrial clumps displaced to one side of the yeast cell (Fig. 1B). The VgrG4 RTD region was sufficient and necessary to cause this effect, as the truncated versions tested bearing the RTD, GST-VgrG4_518-899_ and GST-VgrG4_518-837_ were as efficient as full-length VgrG4 provoking such effect, while a version deleted in the 518-837 region, named ΔRTD, caused no mitochondrial condensation (Fig. 1B). Moreover, toxicity of VgrG4 in yeast was partially abrogated by deletion of the RTD stretch (Supplementary Fig. 1B). The different effect on mitochondria or growth did not reflect differences in expression or stability among the fusions, as determined by immunoblot (Supplementary Fig. 1C).

The mitochondria phenotype was reminiscent of that observed in a *dnm1*Δ mutant, lacking the mitochondrial dynamin-related GTPase Dnm1 (called Drp1 in mammals), in which mitochondria have been reported to collapse into one or more structures and nets^14^. Dnm1 is essential for proper mitochondrial fission in yeast. Dnm1 is recruited to the mitochondrial outer membrane (MOM), where it associates with Fis1, which is anchored to the MOM^15–17^. Once on the membrane Dnm1 triggers the division of the mitochondrial tubular structure^18,19^. As shown in Supplementary Fig. 1D, the condensed mitochondria phenotype of *dnm1* and *fis1* mutants was identical to that of cells expressing VgrG4. Moreover, VgrG4 expression did neither prevent nor aggravate the phenotype of *dnm1* or *fis1* deletants. The lack of an epistatic relation suggested that VgrG4 effect in the cell might involve the mitochondrial fission machinery.

### VgrG4 colocalises with the yeast ERMES component Mdm34 through its VgrG domain

A number of studies have revealed a close contact between the mitochondria and ER^20,21^, and that interactions with the ER promote mitochondrial fission preferentially at the contact sites of the organelles^22^. To assess if VgrG4 associates with and affects ER-mitochondria interactions, we analysed the subcellular localization of VgrG4 in relation to both the ER and the mitochondria. We constructed GFP fusions of full-length VgrG4, its VgrG domain alone (VgrG4_1-517_) and its C-terminal half including the RTD (VgrG4_518-899_). These fusions were efficiently expressed in yeast, as determined by immunoblot (Supplementary Fig. 1E). Ilv6-mCherry and DsRed-HDEL were used as a mitochondrial and ER markers, respectively. Fluorescence microscopy revealed that both GFP-VgrG4 and VgrG4_1-517_ localised at discrete cytoplasmic puncta (Fig. 1C–E and Supplementary Fig. 1F). Relative to mitochondria, full-length VgrG4 puncta were interspersed in the condensed mitochondrial mesh, whereas the puncta of the VgrG N-terminal domain alone were distributed along the cell, often adjacent or to the intact mitochondrial network (Fig. 1C). GFP-VgrG4_518-899_ was found at larger and more diffuse irregular areas, often in proximity to the pole where the bud was emerging in the mother cell (Fig. 1C–E and Supplementary Fig. 1F). Such GFP-VgrG4_518-899_ areas were enclosed by mitochondrial membranes but did not overlap (Fig. 1C). Relative to the ER, GFP-VgrG4 and VgrG4_1-517_ colocalised with ER structures, often different to the perinuclear ER (nER) and cortical ER (cER) (Fig. 1D). The diffuse areas where GFP-VgrG4_518-899_ accumulated were also diffusely stained by the DsRed-HDEL marker, suggesting that they may derive from cytoplasmic ER membranes (Fig. 1D). Collectively, these findings indicate proximity of VgrG4 with the ER and the mitochondria. Furthermore, the non-toxic N-terminal VgrG domain is responsible for the formation of tight puncta, whereas its elimination leads to the toxic RTD domain to mark larger areas, still in the proximity of both mitochondria and ER membranes (Fig. 1C, D).

In *S. cerevisiae*, mitochondria are linked to the ER through the ERMES complex, which supports inter-organellar exchange of Ca^2+^ and lipids, and is essential for Dnm1-triggered mitochondrial fission^23^. The ERMES complex consists of four core components, which include the ER membrane protein Mmm1, and the MOM protein Mdm34^24^. We sought to determine whether VgrG4 co-localised with ERMES components. Thus, we co-expressed GFP-VgrG4, GFP-VgrG4_1-517_ or GFP-VgrG4_518-899_ with Mdm34-DsRed and observed that VgrG4 and GFP-VgrG4_1-517_ puncta tightly co-localised with Mdm34 (Fig. 1E; 89.8% ± 3.8 of GFP-VgrG4 spots and 87.1% ± 1.3 spots overlapped Mdm34-DsRed spots). The more diffuse GFP-VgrG4_518-899_ also colocalised with Mdm34, but only partially (Fig. 1E; 40.0% ± 3.0). In contrast, as shown in (Supplementary Fig. 1F), VgrG4 versions only partially co-localised with Mmm1-DsRed, which in our hands led to a more dispersed cytoplasmic localization as compared to that of Mdm34, suggesting that Mmm1 overexpression from a plasmid led to localisations alternative to the ERMES. Still, most VgrG4 and VgrG4_1-517_ were adjacent to these Mmm1 disperse structures, and some VgrG4_518-899_ spots overlapped. To validate independently the colocalisation of VgrG4 with Mdm34, we performed GST pull-down experiments. For this purpose, we constructed an HA-tagged version of Mdm34 and pulled it down with GST fusions of VgrG4, VgrG4_1-517_ and VgrG4_518-899_. Interestingly, Mdm34-HA co-purified with all three GST-VgrG4 versions (Fig. 1F).

In sum, our results in the yeast model uncover that VgrG4 interferes with mitochondrial morphology via its RTD domain, and that VgrG4 colocalises with the yeast ERMES component Mdm34, mainly through its VrgG domain.

### VgrG4 is translocated into human lung epithelial cells and induces mitochondria fragmentation

**Fig. 2:**
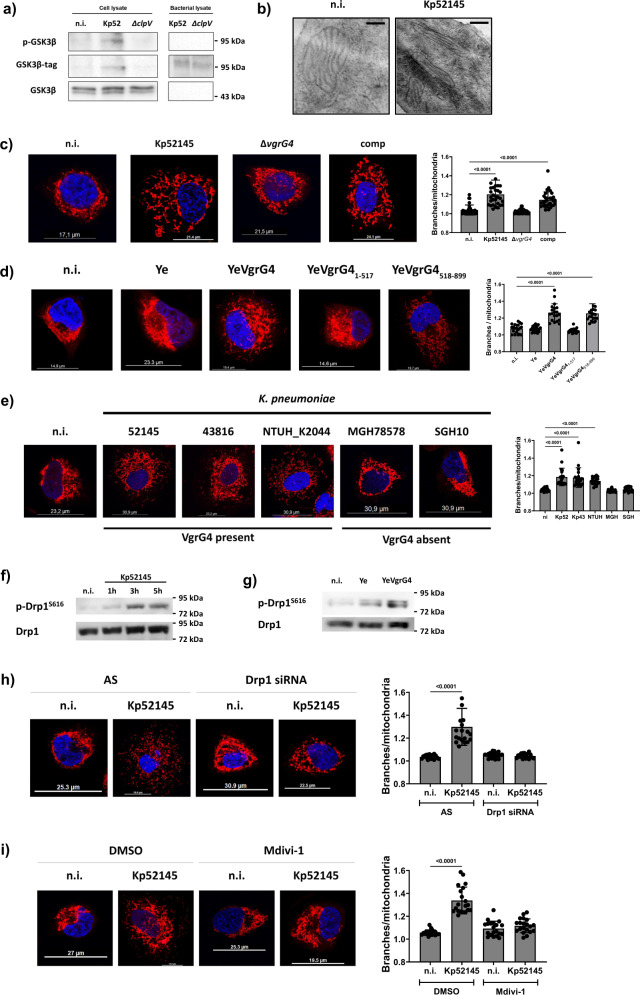
VgrG4 is translocated into A549 cells in a T6SS-dependent manner and induces Drp1 dependent mitochondria fragmentation. Translocation of VgrG4 into lung epithelial cells was analysed by immunoblot of phosphorylated GSK3β, GSK3β-tag and total GSK3β levels (**a**) in lysates of A549 cells infected with Kp52145 (Kp52) and the T6SS inactive *clpV* mutant (strain Δ*clpV*) for 2 hours or left uninfected (n.i.). Bacterial lysates (1 × 10^6^ bacteria) were run for the same antibodies. Mitochondria ultrastructure was analysed by transmission electron microscopy (TEM) (**b**) in A549 cells infected with Kp52145 for 5 hours or left uninfected (n.i.) (magnification 20,000×, scale bar 200 nm). Mitochondria fragmentation (**c**) was analysed by confocal microscopy of A549 cells treated with mitotracker red (50 μM, 30 min before infection, in red) and infected for 3 h with Kp52145, the *vgrG4* mutant (Δ*vgrG4*), or the complemented strain (comp, Δ*vgrG4*/pBAD30*vgrG4*) or left uninfected (n.i.). The number of branches/mitochondria was determined with the Mitochondria Analyzer plugin for ImageJ. The graph is the result of the analysis of twenty images per condition of three independent experiments representing average with standard deviation error bars; at least 100 cells were analysed per sample. Fragmentation was also analysed following infection with different *Y. enterocolitica* strains for 90 min (**d**) and following infection with different wild-type *K. pneumoniae* strains for 3 h (**e**); at least 100 cells were analysed per sample. Nuclei were stained with Hoechst (DAPI, in blue). All images and immunoblots are representative of three independent experiments. **f** Immunoblot analysis of the levels of phosphorylated Drp1 (S616) and total Drp1 in lysates of A549 cells infected with Kp52145 for the indicated times. Phosphorylation of Drp1 was also analysed in lysates of A549 cells infected with *Y. enterocolitica* (Ye) and YeVgrG4 for 90 min (**g**). **h** Confocal microscopy of A549 cells transfected with a non-silencing control (AS – All Stars) or with a Drp1 siRNA, and treated with mitotracker red (50 μM, 30 min, in red), and infected with Kp52145 for 3 h. To confirm further the role of Drp1 in VgrG4-induced mitochondria fragmentation, cells were treated with the Drp1 inhibitor Mdivi-1 (10 μM, 2 h before infection) or DMSO (vehicle solution) and infected with Kp52145 for 3 h (**i**); at least 100 cells were analysed per sample. One-way ANOVA with Tukey’s test for multiple comparison was applied for statistical significance for all the indicated comparisons (*p* values indicated in the graph).

We next sought to establish whether VgrG4 could also target mitochondria in mammalian cells. We focused on human lung cells as they form the first line of defence against infections during pneumonia. *K. pneumoniae* does not invade these cells^25,26^ and, therefore, we asked whether *K. pneumoniae* injects VgrG4 into the cells in a T6SS-dependent manner. To address this question, VgrG4 was tagged with a 13-residue phosphorylatable peptide tag (GSK) derived from the human GSK-3β kinase^27^. Translocation of a GSK-tagged protein into a eukaryotic cell results in host cell protein kinase-dependent phosphorylation of the tag, which can be detected with phospho-specific GSK3β antibodies^27^. Likewise other pathogens^27^, Kp52145 did not phosphorylate the GSK-tag (Fig. 2A). Infection of A549 cells with Kp52145 expressing VgrG4-GSK resulted in the translocation of the protein that was subsequently phosphorylated (Fig. 2A). Translocation of VgrG4-GSK was T6SS-dependent because we could not detect any phosphorylated VgrG4 in cells infected with the *clpV* mutant expressing VgrG4-GSK (Fig. 2A). A *clpV* mutant cannot assemble a functional T6SS^12^.

We next investigated the ultrastructural changes of the mitochondria after *Klebsiella* infection using transmission electron microscopy (TEM). Non-infected cells exhibited mitochondria with normal-appearing cristae (Fig. 2B). In contrast, in *Klebsiella* infected cells mitochondria were elongated with increase surface area (Fig. 2B). This phenotype is indicative of mitochondria fragmentation. Confocal microscopy experiments showed that Kp52145 induced mitochondrial network fragmentation in A549 cells (Fig. 2C). Similar phenotype was observed in cells treated with carbonyl cyanide m-chlorophenylhydrazone (CCCP) (Supplementary Fig. 2); this drug triggers mitochondria fragmentation^28–30^. In contrast, the phenotype was not observed in cells infected with the *vgrG4* mutant (Fig. 2C). Complementation of *vgrG4* restored the fragmentation of the mitochondrial network, demonstrating that the phenotype is VgrG4 dependent. The mitochondrial network was analysed using a semi-automated ImageJ plug^31^. The mitochondrial network of Kp52145-infected cells presented higher number of branches and junctions than non-infected cells or cells infected with the *vgrG4* mutant, which were indistinguishable (Fig. 2C and Supplementary Fig. 3A). The total length of the branches was reduced in Kp52145-infected cells compare to non-infected cells or cells infected with the *vgrG4* mutant (Supplementary Fig. 3A). Complementation of the mutant restored the mitochondrial network (Supplementary Fig. 3A). No differences were found on the mean length diameter of the branches (Supplementary Fig. 3A). VgrG4-induced mitochondrial fragmentation was also observed in human alveolar primary lung NuLi-1 cells (Supplementary Fig. 3B), indicating that the phenotype was not cell line dependent.

Because VgrG proteins are also known to act as cargo for other T6SS effectors^8^, the possibility exists that the mitochondrial fragmentation could be mediated by other T6SS effector(s) delivered by VgrG4. To exclude this possibility, we took advantage of a *Yersinia* toolbox which allows to study the cellular effects of single bacterial effectors following *Yersinia* type 3 secretion system (Ysc)-T3SS-mediated injection into eukaryotic cells^32^. Control experiments confirmed the secretion of VgrG4 tagged with a VSV-G epitope in conditions in which the T3SS is active (Supplementary Fig. 3C). Cells infected with *Y. enterocolitica* encoding *vgrG4* (hereafter YeVgrG4) presented fragmentation of the mitochondrial network in contrast to cells infected with the *Y. enterocolitica* control strain (Fig. 2D). Analysis of the mitochondrial network showed that YeVgrG4 induced similar changes as those triggered by Kp52145 (Fig. 2D). The assessment of truncated variants of VgrG4 confirmed that the C-terminal region containing the RTD domain was essential and sufficient to trigger the fragmentation of the mitochondrial network (Fig. 2D).

Recently, we have demonstrated that there is considerable diversity in the T6SS loci amongst *K. pneumoniae* strains^12^. We reasoned that strains encoding *vgrG4* should trigger the fragmentation of the mitochondrial network similarly to Kp52145. In good agreement, *vgrG4* positive strains, ATCC43816 and NTUH-K2044, induced the fragmentation of mitochondria whereas this was not the case for the *vgrG4* negative strains SGH10 and MGH78578 (Fig. 2E). Analysis of the mitochondrial network revealed that only *vgrG4* positive strains induced similar changes as those observed in cells infected with Kp52145 (Fig. 2E).

Collectively, these results demonstrate that *K. pneumoniae* induces the fragmentation of the mitochondrial network in human lung epithelial cells upon T6SS-dependent injection of VgrG4.

### VgrG4-induced mitochondrial fragmentation is Drp1-dependent

Mitochondrial fragmentation could result from enhanced fission^33^. The GTPase dynamin-related protein 1 (Drp1) is critically essential for mitochondrial fission^34^. Phosphorylation of Drp1 plays a crucial role in its activity with Ser616 phosphorylation promoting fission^35^. We therefore investigated whether VgrG4 triggers the phosphorylation of Drp1. Immunoblotting experiments showed that Kp52145 and YeVgrG4 induced the phosphorylation of Drp1 (Fig. 2F, G, respectively), indicating that VgrG4 was sufficient to trigger the activation of Drp1. The quantification of the blots is shown in (Supplementary Fig. 4). To determine whether VgrG4-induced mitochondrial fragmentation is Drp1-dependent, we infected cells in which *drp1* was silenced by siRNA. Kp52145-induced mitochondrial fragmentation was not observed in *drp1* knockdown cells in contrast to cells treated with All Stars control siRNA (Fig. 2H). *drp1* knockdown efficiency was higher than 60% (Supplementary Fig. 5A, B). To substantiate further the role of Drp1, we used Mdivi-1, a compound that inhibits Drp1 function during mitochondrial fission^36^ although some caveats on the specificity of the inhibitor have been reported^37^. When Mdivi-1-treated cells were infected with Kp52145, we did not observe any mitochondrial fragmentation (Fig. 2I). As anticipated, YeVgrG4-triggered mitochondrial fragmentation was not detected in Mdivi-1-treated cells (Supplementary Fig. 3D), and in cells in which *drp1* was silenced by siRNA (Supplementary Fig. 3E). Altogether, our results show that VgrG4-induced fragmentation of mitochondria depends on the activity of Drp1.

### VgrG4-induced mitochondrial fragmentation does not impair mitochondria physiology

We next investigated the physiology of the mitochondria in *Klebsiella*-infected cells. We first assessed whether VgrG4-induced mitochondrial fragmentation affects the contribution of the mitochondria to cellular bioenergetics using a Seahorse XFe96 analyser. The oxygen consumption rate (OCR) increased following infection with Kp52145 (Supplementary Fig. 6A), indicating an increase in mitochondrial basal respiration (Supplementary Fig. 6B). Addition of oligomycin triggered a decrease of cellular OCR, however the OCR was still significantly higher in Kp52145 infected cells compared to non-infected ones indicating that not all oxygen consumption is used for ATP production in *K. pneumoniae*-infected cells (Supplementary Fig. 6A, B). ATP levels were higher in Kp52145-infected cells than in non-infected cells (Supplementary Fig. 6B). The fact that the proton leak was not different between non-infected cells and Kp52145-infected ones indicates no differences in the basal respiration not coupled to ATP production (Supplementary Fig. 6B). Subsequent addition of FCCP, which stimulates respiration, showed that the maximal respiration capacity was similar between non-infected and Kp52145-infected cells (Supplementary Fig. 6A, B). The spare respiratory capacity was not significantly different between Kp52145-infected cells and non-infected ones (Supplementary Fig. 6A, B), indicating that *K. pneumoniae* infection does not deplete the cellular energy via an increased oxidative phosphorylation. The fact that OCR was higher in Kp52145-infected cells as compared to non-infected ones after the addition of rotenone and antimycin A indicates that the non-mitochondrial respiration is increased in *K. pneumoniae*-infected cells (Supplementary Fig. 6A, B). Altogether, these findings demonstrate that Kp52145 infection does not impair the mitochondria bioenergetics. The fact that the wild-type, the *vgrG4* mutant and the complemented strains triggered similar changes (Supplementary Fig. 6A, B) demonstrates that *K. pneumoniae*-triggered metabolic changes are not affected by VgrG4-induced fragmentation of the mitochondrial network.

The release of mtDNA to the cytosol of cells has been associated with the fragmentation of mitochondria^38^. Therefore, we assessed whether VgrG4 will trigger the release of mtDNA to the cytosol by determining the cytoplasmic levels of the mitochondria-encoded cytochrome c oxidase I gene (mt-CO1). qPCR experiments showed that YeVgrG4 infection resulted in the presence of mtDNA in the cytosol of A549 and NuLi-1 cells (Supplementary Fig. 7A, B, respectively). The presence of mtDNA in the cytosol results in the activation of the cGAS-STING signalling pathway leading to the upregulation of the expression of type I IFN and interferon-stimulated genes (ISGs)^38^. To investigate whether VgrG4-induced release of mtDNA activates the STING signalling pathway, we challenged NuLi-1 cells and measured transcription of target genes. A549 cells do not express STING^39^ and, therefore, they are not suitable for this analysis. As we expected, YeVgrG4 induced the expression of *ifnb*, and the ISGs *irf3*, *isg15* and *isg54* in NuLi-1 cells (Supplementary Fig. 7C). However, this was not the case in cells treated with the STING antagonist H151^40^. Combined this suggests that VgrG4 induces mtDNA release which stimulates STING-governed signalling.

Mitochondrial fragmentation and release of mtDNA have been linked to the dissipation of the mitochondria membrane potential ΔΨ, important for the generation of ATP, transport across the mitochondria membrane^41^, and cell death^38^. However, we found that neither infection with *K. pneumoniae* nor with YeVgrG4 caused any change in ΔΨ, in contrast to cells treated with the membrane uncoupler CCCP (Supplementary Fig. 7D). In line with this, we observed no differences in the levels of intracellular ATP between non-infected cells, and those infected with *K. pneumoniae* or YeVgrG4 (Supplementary Fig. 4E). We also observed no differences in cell viability between non-infected and infected cells (Supplementary Fig. 7F), finding consistent with the lack of change in the ΔΨ.

Altogether, these data suggest that VgrG4-induced mitochondrial fragmentation results in the release of mtDNA to the cytosol but without perturbation of the functionality of mitochondria.

### VgrG4 colocalises with the ER protein mitofusin 2

**Fig. 3:**
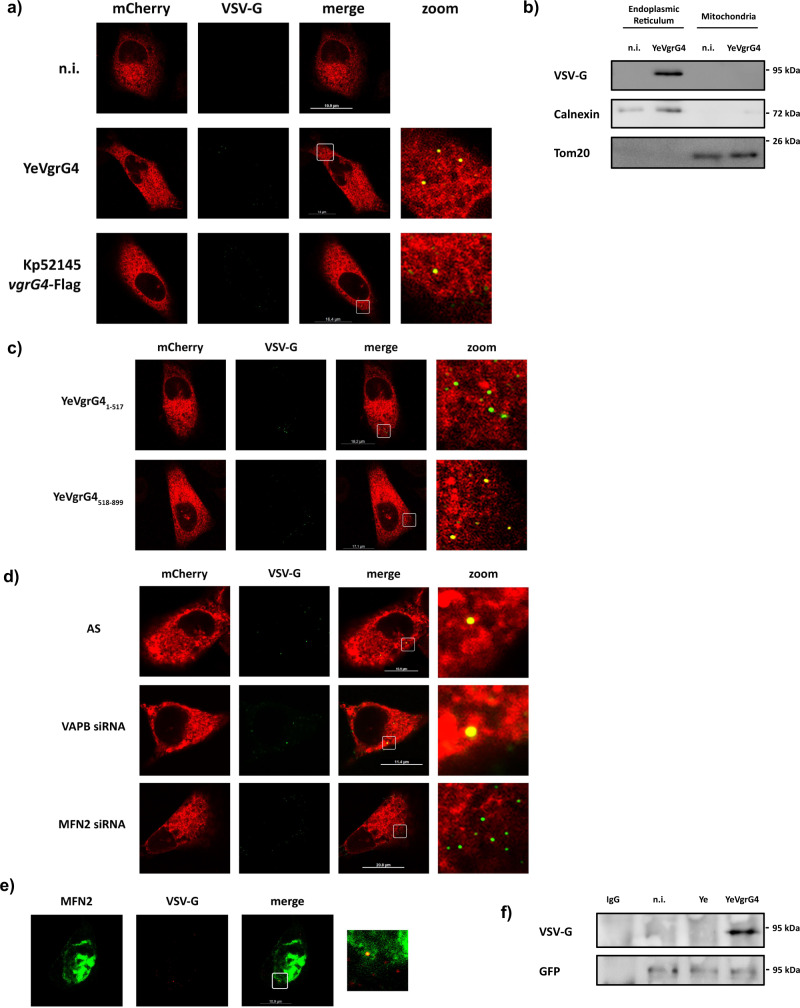
VgrG4 colocalizes with the ER. VgrG4 localisation within the cells was analysed by confocal microscopy of A549 mCherryER cells (ER in red). Cells were infected with YeVgrG4 for 90 min, or Kp52145 for 3 h, or left uninfected (n.i.) VgrG4 was stained with VSV-G or FLAG (in green) (**a**). **b** Immunoblot analysis of VgrG4-tagged with VSV-G, Calnexin (ER marker) and Tom20 (mitochondria marker) in fractionated lysates of A549 cells infected with YeVgrG4 for 90 min or left uninfected (n.i.). **c** Confocal microscopy of A549 mCherryER cells infected with *Y. enterocolitica* encoding N- and C-terminal truncated forms of VgrG4 tagged with VSV-G for 90 min. To determine the tethering protein associating with VgrG4, A549 mCherryER cells were transfected with siRNAs for VAPB or MFN2 and infected with YeVgrG4 for 90 min (**d**). **e** Confocal microscopy of A549 cells transfected with the MFN2-YFP plasmid (MFN2 in green) and infected with YeVgrG4 for 90 min. VgrG4 was stained with VSV-G. **f** Immunoblot analysis of VSV-G (VgrG4) and GFP (MFN2) levels in immunoprecipitates of A549. 24 h after transfection with a MFN2-YFP plasmid, cells were infected with YeVgrG4 for 90 min. Lysates were immunoprecipitated using anti-GFP antibody, and membranes were first probed with antibody against VSV-G and subsequently with antibody against GFP. Pre-immune mouse IgG served as negative control. All images and immunoblots are representative of three independent experiments.

We next sought to establish the colocalisation of VgrG4 within mammalian cells. VgrG4 delivered by *Yersinia* showed a puncta distribution within A549 cells; however these puncta did not colocalise with mitochondria although they were found in closed proximity (Supplementary Fig. 8). The colocalisation of VgrG4 with the ER in yeast led us to examine whether VgrG4 colocalises with the ER in mammalian cells. Confocal microscopy experiments revealed the colocalisation of VSV-G tagged VgrG4 puncta with the ER (Fig. 3A). FLAG-tagged VgrG4 injected by Kp52145 also showed a puncta distribution, and we observed its colocalisation with the ER (Fig. 3A). Fractionation experiments corroborated that *Yersinia*-injected VgrG4 was present in the ER but not in the mitochondria fraction (Fig. 3B). The VgrG4 C-terminal region containing the RTD domain was sufficient for VgrG4 colocalisation with the ER (Fig. 3C) whereas this was not observed in cells injected with the N-terminal region of VgrG4 (Fig. 3C).

In yeast, VgrG4 colocalised with ERMES component Mdm34. However, no mammalian homologue exists of the ERMES components. Instead, a number of tethering proteins have been reported to maintain the ER-mitochondria MCS, including vesicle-associated membrane protein B (VAPB), protein tyrosine phosphatase-interacting protein-51 (PTPIP51), Mitofusin (Mfn) 1/2, inositol 1,4,5-trisphosphate receptor (IP3R), glucose-regulated protein 75 (GPR75), voltage-dependent anion channel (VDAC1) complex, and B-cell receptor-associated protein 31 (Bap31)^42^. Recent work strongly indicates that VAPB and Mfn2 are the main ER-mitochondria tethering proteins^43^. To determine whether these proteins are needed for the colocalisation of VgrG4 with the ER, their expression was reduced by siRNA and the colocalisation of VgrG4 with the ER assessed by confocal microscopy. Control experiments confirmed the knockdown of VAPB and Mfn2 (Supplementary Fig 5). Figure 3D shows that the colocalisation of VgrG4 with the ER was lost only in cells deficient for Mfn2. Furthermore, in cells transfected with an YFP fusion of Mfn2, we detected the colocalisation of Mfn2-YFP with VgrG4 by confocal microscopy (Fig. 3E). Moreover, Mfn2-YFP co-immunoprecipitated VSV-G tagged VgrG4 (Fig. 3F).

Altogether, these results establish that VgrG4 colocalises with the ER protein Mfn2. The C-terminal region containing the RTD domain mediates the colocalisation of VgrG4 with the ER and Mfn2.

### VgrG4 triggers the transfer of Ca^2+^ to the mitochondria from the ER

**Fig. 4:**
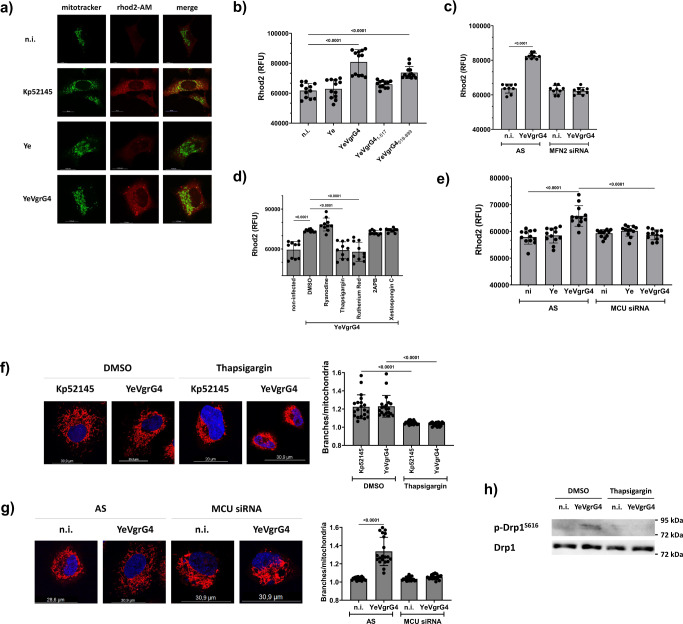
VgrG4 triggers the transfer of Ca^2+^ from the ER to the mitochondria causing fragmentation. **a** Confocal microscopy of A549 cells treated with mitotracker green (50 μM, 30 min before infection, in green) and then infected with *Y. enterocolitica* strains for 90 min, or with Kp52145 for 3 h. After 90 min or 3 h, Rhod2 in HBSS without calcium (50 µM, in red) was added for 30 min to stain the calcium accumulation in the mitochondria. **b** Rhod2 fluorescence was measured at 90 min post infection as indicated before in cells infected with *Y. enterocolitica* strains encoding truncated versions of VgrG4. Rhod2 accumulation was measured in cells knockdown for MFN2 (20 nM) by siRNA transfection and infected with YeVgrG4 (**c**). Cells were treated with ryanodine (100 nM), thapsigargin (1 μM), ruthenium red (100 μM), 2APB (10 μM) or Xestospogin C (10 μM) or treated with vehicle control (DMSO, no inhibitor) 60 min post infection. Rhod2 fluorescence was measured as previously described (**d**). **e** Rhod2 fluorescence was measured in cells transfected with siRNA for the mitochondria calcium uniporter (MCU) and infected for 2 h as described in **b**. **f** Confocal microscopy of A549 cells treated with mitotracker red (50 μM, 30 min, in red) and infected with YeVgrG4 for 90 min or Kp52145 for 3 h. Nuclei were stained with Hoechst (DAPI, in blue), and at least 100 cells were analysed per sample. Cells were treated with thapsigargin (1 μM, 30 min before end of infection) or DMSO (vehicle solution). Mitochondria fragmentation was assessed by confocal microscopy in cells transfected with siRNA for MCU (20 nM) or AS control and infected with YeVgrG4 (**g**); at least 100 cells were analysed per sample. The levels of S616 phosphorylated Drp1 and tubulin were determined by immunoblotting in lysates of YeVgrG4-infected A549 cells treated with thapsigargin (1 μM) or vehicle control (DMSO) for 30 min before the end of the infection (**h**). One-way ANOVA with Tukey’s multiple comparison test was applied for statistical significance (*p* values indicated in the graph). Images are representative of three independent experiments. Data in graphs are presented as the mean ± SD of five independent experiments measured in duplicate.

The ER-mitochondria contact sites are crucial in the regulation of inter-organellar Ca^2+^ exchange^44^. We examined whether VgrG4 affects the transfer of Ca^2+^ from the ER to the mitochondria. To detect the accumulation of Ca^2+^in the mitochondria, we used the cell permeable mitochondria probe Rhod-2 AM whose fluorescence increases upon binding Ca^2+^. Kp52145 infection increased the fluorescence of Rhod-2 in the mitochondria (Fig. 4A). VgrG4 was sufficient to evoke the accumulation of Ca^2+^in the mitochondria as YeVgrG4 infection also increased Rhod-2 fluorescence (Fig. 4A). Time course experiments showed an increase in Rhod-2 fluorescence over time following YeVgrG4 infection (Supplementary Fig. 9A), being the RTD containing domain of VgrG4 sufficient to increase the fluorescence of Rhod-2 (Fig. 4B). The colocalisation of VgrG4 with the ER Mfn2 is essential to evoke the Ca^2+^ accumulation in the mitochondria because no increase of Rhod-2 fluorescence was found in *mfn2* knockdown cells by siRNA (Fig. 4C). Others have shown that silencing of *mfn2* does not affect agonist-induced ER Ca^2+^ release^45^.

To determine the receptors and channels responsible for VgrG4-induced accumulation of Ca^2+^ in the mitochondria, we took advantage of a panel of well-established inhibitors affecting ER-mitochondria Ca^2+^ exchange. Blocking of ryanodine receptors using ryanodine had no effect on Ca^2+^ accumulation following infection with YeVgrG4 (Fig. 4D). Likewise, blocking of IP3-mediated Ca^2+^ release with Xestospongin C or inhibition of both IP_3_ receptors and TRP channels with 2-Aminoethoxydiphenyl borate (2APB) had no effect on Rhod-2 fluorescence (Fig. 4D). On the contrary, thapsigargin, a non-competitive inhibitor of the sarco/endoplasmic reticulum Ca-ATPase (SERCA), abrogated YeVgrG4-mediated increase in Rhod-2 fluorescence (Fig. 4D), demonstrating that VgrG4-induced Ca^2+^ accumulation in the mitochondria is dependent on the activation of the ER SERCA pumps. The fact that the inhibitor ruthenium red decreased VgrG4-induced increase of Rhod-2 fluorescence (Fig. 4D) indicates that mitochondria Ca^2+^ accumulation is dependent on the mitochondrial calcium uniporter complex (MCU). Further substantiating this result, silencing of *mcu* with siRNA resulted in no increase in Rhod-2 fluorescence following YeVgrG4 infection (Fig. 4E).

Collectively, these findings demonstrate that VgrG4 triggers the accumulation of Ca^2+^ in the mitochondria via the mitochondria calcium uniporter complex upon activation of the ER SERCA pumps.

### VgrG4-induced Ca^2+^ accumulation in the mitochondria mediates the fragmentation of the mitochondria network

Ca^2+^ levels have been implicated in the regulation of mitochondria morphology^46^. Therefore, we sought to establish whether VgrG4-induced Ca^2+^ accumulation in the mitochondria results in the fragmentation of the mitochondria network. Confocal microscopy experiments uncovered that inhibition of the ER SERCA pumps with thapsigargin abrogated YeVgrG4 and Kp51245-induced mitochondrial fragmentation (Fig. 4F) whereas the inhibition of ryanodine receptors, IP_3_ receptors and TRP channels with ryanodine, Xestospongin C, and 2APB, respectively had no effect on YeVgrG4-triggered fragmentation of the network (Supplementary Fig. 9B). YeVgrG4 did not affect the mitochondria network in cells in which *mcu* was silenced using siRNA (Fig. 4G). Moreover, treatment of cells with thapsigargin also abrogated YeVgrG4-induced phosphorylation of Drp1 (Fig. 4H and Supplementary Fig. 4), uncovering that the transfer of Ca^2+^ to the mitochondria is the signal triggering the activation of Drp1 in VgrG4-treated cells.

To sustain further that Ca^2+^ release from the ER is sufficient to induce the fragmentation of the mitochondria, cells were treated with carbachol that triggers the release of Ca^2+^ from the ER^47^. Control experiments showed that carbachol treatment increased the fluorescence of Rhod-2 (Supplementary Fig. 10A). Carbachol treatment resulted in the fragmentation of the mitochondrial network (Supplementary Fig. 10B), and the phosphorylation of Drp1 (Supplementary Fig. 10C). The fact that treatment of cells with the Drp1 inhibitor Mdivi-1 abrogated carbachol-induced mitochondria fragmentation (Supplementary Fig. 10B) demonstrates that carbachol-induced fragmentation is Drp1-dependent. Therefore, carbachol treatment recapitulates VgrG4 action.

Altogether, these results strongly support the notion that VgrG4-induced mitochondrial Ca^2+^ accumulation from the ER mediates the activation of Drp1 responsible for VgrG4-induced fragmentation of the mitochondria network.

### VgrG4 induces mitochondria ROS upon activation of the innate receptor NLRX1

**Fig. 5:**
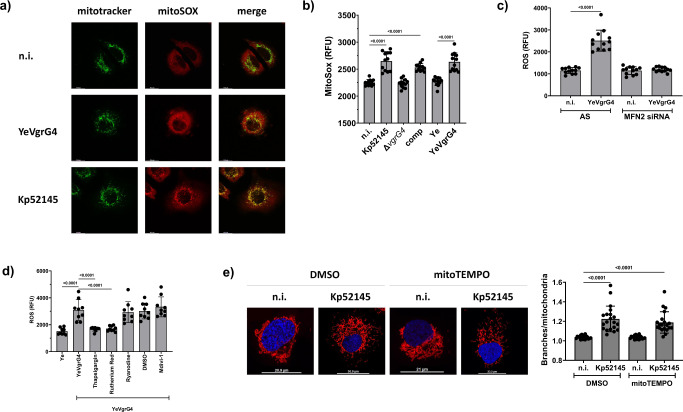
VgrG4 induces mitochondrial ROS production. **a** Mitochondrial ROS (mtROS) was detected in infected A549 cells by confocal microscopy. Cells were treated with mitotracker green (50 μM, 30 min, in green), infected with YeVgrG4 for 90 min or with Kp52145 for 3 h, and treated with mitoSOX red mitochondrial superoxide indicator (10 µM for 30 min incubated in calcium free HBSS). Fluorescence was measured (**b**) in A549 cells upon infection with *Y. enterocolitica* strains for 90 min or *K. pneumoniae* strains for 3 h, followed by incubation with mitoSOX (10 µM incubated in calcium free HBSS for 30 min prior to measurement). **c** To demonstrate that VgrG4 localisation is important for ROS production, 2’,7’-dichlorofluorescein (DCF) was used to measure ROS production by A549 cells transfected with MFN2 siRNA or non-silencing (AS) control and infected with YeVgrG4 for 90 min. **d** DCF fluorescence was measured in A549 cells pre-treated with Mdivi-1 (10 µM, 2 h) or vehicle control (DMSO), and infected with YeVgrG4 for 90 min. Cells were treated with thapsigargin (1 μM), ruthenium red (100 μM), ryanodine (100 nM) 30 min before the end of infection. Mitochondria fragmentation was analysed by confocal microscopy of A549 cells treated with mitotracker red (50 μM, 30 min, in red) and stained with Hoechst (in blue). Cells were pre-treated with mitoTEMPO, a mitochondrial superoxide scavenger (10 μM, 2 h), and infected with Kp52145 for 3 h (**e**); at least 100 cells were analysed per sample. One-way ANOVA with Tukey’s multiple comparison test was applied for statistical significance (*p* values indicated in the graph).

Mitochondria produce ROS (mtROS) due to electron slippage during oxidative phosphorylation^48^, and there are indications of a crosstalk between Ca^2+^ levels in the mitochondria and mtROS production^44^. We then investigated whether VgrG4 induces mtROS by assessing the oxidation of the mitochondria localized fluorescent dye mitoSOX. Kp52145 and YeVgrG4 infections resulted in the oxidation of mitoSOX that colocalised with mitochondria (Fig. 5A). Quantification of mitoSOX oxidation corroborated that Kp52145 infection increased the levels of mtROS whereas the *vgrG4* mutant did not increase mtROS levels over those of the non-infected cells (Fig. 5B). Complementation experiments confirmed that mtROS induction is VgrG4-dependent (Fig. 5B). The fact that YeVgrG4 also induced mtROS (Fig. 5A, B) suggests that VgrG4 translocation to cells is sufficient to elicit mtROS. In *mfn2* knockdown cells, YeVgRG4 did not induce mtROS, indicating that the ER colocalization of VgrG4 is essential for the production of mtROS (Fig. 5C).

We next assessed whether VgrG4-induced Ca^2+^ accumulation in the mitochondria mediates mtROS induction. Thapsigargin and ruthenium red, two drugs that inhibited the VgrG4-triggered transfer of Ca^2+^ into the mitochondria (Fig. 4D), abrogated YeVgrG4-induced mtROS (Fig. 5D). In contrast, ryanodine, an inhibitor that did not affect VgrG4-triggered Ca^2+^ accumulation in mitochondria (Fig. 4D), did not reduce VgrG4-induced mtROS (Fig. 5D). Carbachol treatment elicited mtROS (Supplementary Fig. 10D), substantiating that the accumulation of Ca^2+^ into the mitochondria induces mtROS. Altogether, these results support the notion that VgrG4-stimulated Ca^2+^ accumulation is responsible for mtROS levels. However, VgrG4-induced mtROS is independent of the activation of Drp1 because treatment of cells with Mdivi-1 had no effect on YeVgrG4-mediated oxidation of mitoSOX (Fig. 5D).

We next sought to provide mechanistic insights into the molecular basis connecting VgrG4-induced Ca^2+^ accumulation and mtROS. NLRX1 is a mitochondrial NOD-like receptor that has been suggested to interact with the electron transport chain and potentiate the production of mtROS^49^. Therefore, we asked whether VgrG4-induced mtROS is dependent on NLRX1 using the oxidation of the fluorescence dye dichlorofluorescein (DCF) as read-out. Supplementary Fig. 11A shows that YeVgrG4 induced fluorescence levels of DCF were significantly lower in *nlrx1* knockdown cells using siRNA than in cells treated with non-silencing control All Stars siRNA. Interestingly carbachol did not elicit mtROS in cells in which *nlrx1* was silenced (Supplementary Fig. 10E), demonstrating that the accumulation of Ca^2+^ into the mitochondria triggers mtROS in a NLRX1-dependent manner.

We next questioned whether NLRX1-induced mtROS affects the mitochondria morphology. Scavenging mtROS with mitoTEMPO did not abrogate Kp52145-induced fragmentation of the mitochondria network (Fig. 5E). Reinforcing the notion that ROS had no effect on mitochondria morphology, the potent ROS inducer H_2_O_2_ had no effect on the mitochondria network (Supplementary Fig. 6F). YeVgrG4 still induced the fragmentation of the mitochondria network in *nlrx1* knockdown cells, and the fragmentation was not different to that induced by the infection in non-silencing control All Stars siRNA treated cells (Supplementary Fig. 11B). Control experiments confirmed that there were no differences in the fluorescence of Rhod-2 in All stars treated and *nlrx1* knockdown cells infected with YeVgrG4 (Supplementary Fig. 11C). Altogether, these results are consistent with the notion that VgrG4-induced mtROS does not affect the mitochondria network, and that NLRX1 does not control VgrG4-induced influx of Ca^2+^ to the mitochondria.

Collectively, these data demonstrate that VgrG4-triggered Ca^2+^ accumulation in the mitochondria induces mtROS in a NLRX1-dependent manner.

### NLRX1 elicited ROS mediates cullin 1 deNEDDylation to modulate epithelial signalling

**Fig. 6:**
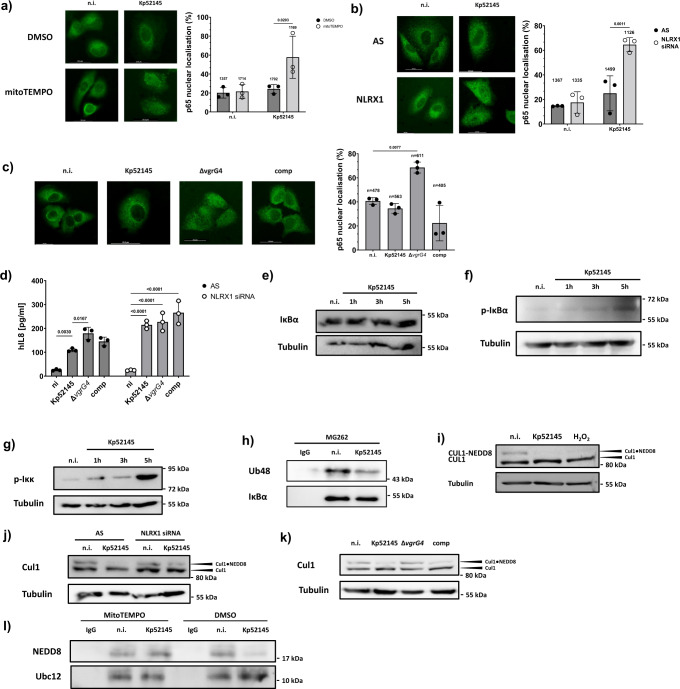
NLRX1-mediated ROS controls NF-κB signalling and affects cullin 1 NEDDylation. Immunofluorescence microscopy of A549 cells stained with antibody for the p65 NF-κB subunit. Cells were pre-treated with mitoTEMPO (10 μM, 2 h pre-infection), or with vehicle control (DMSO) and infected with Kp52145 for 3 h (**a**). **b** Cells were transfected with NLRX1 siRNA (50 nM) or a non-silencing control (AS) and infected with Kp52145 for 3 h. **c** Cells were infected with Kp52145, the *vgrG4* mutant (Δ*vgrG4*) or the complemented strain (comp, Δ*vgrG4*/pBAD30*vgrG4*) for 3 h. The percentage of p65 NF-κB localised in the nucleus is represented on the graph in **a**–**c** and it is the result of counting of minimum of hundred cells from each of three independent experiments. The total number of counted cells is indicated on top of each bar. ELISA of IL-8 secreted by A549 cells transfected with either NLRX1 siRNA (50 nM) or a non-silencing control (AS) and infected with *K. pneumoniae* strains. After 3 h of contact, the medium was replaced with medium containing gentamicin (100 µg/mL) to kill extracellular bacteria, and after 2 h the medium was collected (**d**). Images are representative of three independent experiments. Data in graphs are presented as the mean ± SD of three independent experiments. Two way-ANOVA with Holm-Sidak’s multiple comparisons test was used for statistical significance (*p* values indicated in the graph). **e** Immunoblot analysis of total IκBα and tubulin levels in lysates of A549 cells infected with Kp52145 for the indicated times. **f** Immunoblot analysis of phosphorylated IκBα and tubulin levels in lysates of A549 cells infected with Kp52145 for the indicated times. **g** Levels of phosphorylated Iκκα/β and tubulin in cells infected with Kp52145 for the indicated times. IκBα immunoprecipitation and immunoblot for K48-linkage specific polyubiquitin (Ub48) in cells treated with the proteasome inhibitor MG262 (5 μM, 2 h before infection), and infected with Kp52145 for 3 h or left uninfected (n.i.). Pre-immune mouse IgG was used as a control for immunoprecipitation (**h**). Immunoblot analysis of Cul-1 and tubulin levels in lysates of A549 cells infected with Kp52145 for 5 h, or treated with H_2_O_2_ (5 μM, 5 min). Cul-1 appears as a doublet, with the higher molecular band representing the NEDDylated form of Cul-1 (**i**). **j** Immunoblot analysis of Cul-1 and tubulin levels in lysates of A549 cells transfected with NLRX1 siRNA (50 nM) or a non-silencing control (AS), infected with Kp52145 for 5 h. **k** Cells were infected with Kp52145, the *vgrG4* mutant (Δ*vgrG4*) or the complemented strain (comp) for 5 h, and the levels of Cul-1 and tubulin assessed by western blot. **l** Cells were pre-treated with mitoTEMPO (10 μM, 2 h pre-infection) or a vehicle control (DMSO), infected with Kp52145 for 5 h, and Ubc12 was immunoprecipitated followed by detection of NEDD8 and Ubc12 levels by immunoblotting. Images are representative of three independent experiments.

Extracellular commensal bacteria exploit ROS to attenuate the activation of the immunoregulatory NF-κB pathway^50^ that governs host defence pathways against pathogens including *K. pneumoniae*^25,51–53^. We hypothesized that *Klebsiella* exploits NLRX1-induced mtROS to limit the activation of NF-κB. In cells pre-treated with mitoTEMPO we observed an increase in the nuclear translocation of the NF-κB p65 unit following infection with Kp52145 (Fig. 6A). A similar result was obtained following infection of *nlrx1* knockdown cells (Fig. 6B). These results suggested that VgrG4 limits the activation of NF-κB. Indeed, and in sharp contrast to the wild-type strain, the *vgrG4* mutant induced the nuclear translocation of p65 (Fig. 6C). Complementation of the mutant restored the wild-type phenotype (Fig. 6C). Moreover, VgrG4 was sufficient to reduce the p65 translocation induced by *Y. enterocolitica* (Supplementary Fig. 12A). However, this was not the case in cells pre-treated with mitoTEMPO or in cells knockdown for *nlrx1* (Supplementary Fig. 12B and 12C, respectively). Consistent with VgrG4-mediated control of NF-κB activation, the *vgrG4* mutant induced higher levels of IL8 than the wild-type strain (Fig. 6D). Moreover, translocation of VgrG4 was sufficient to reduce *Y. enterocolitica*-induced IL8 levels (Supplementary Fig. 12D). No differences between strains were observed in *nlrx1* knockdown cells (Fig. 6D and Supplementary Fig. 12D). Altogether, these results support the notion that VgrG4-induced NLRX1-dependent mtROS negative regulates the activation of NF-κB with a concomitant reduction in inflammation.

We next addressed at which level of the NF-κB signalling cascade VgrG4 exerts its negative effect via NLRX1-induced ROS. In the canonical NF-κB pathway, nuclear translocation of NF-κB is preceded by phosphorylation and subsequent degradation of IκBα by the ubiquitin proteasome^54^. Experiments confirmed that Kp52145 infection did not affect the levels of IκBα (Fig. 6E). However, we noted that infection did result in the phosphorylation of IκBα (Fig. 6F), suggesting that the signalling cascade leading to the phosphorylation of IκBα is not affected by VgrG4. Supporting this notion further, Kp52145 infection resulted in the phosphorylation of the kinase Iκκα/β (Fig. 6G) that controls the phosphorylation of IκBα. These results led us to investigate whether the polyubiquitination of IκBα is affected in *K. pneumoniae*-infected cells. Indeed, we found a decrease in the levels of ubiquitinated IκBα in Kp52145-infected cells (Fig. 6H). Interestingly, this is not specific of IκBα because we also observed a decrease in the levels of ubiquitination of β-catenin (Supplementary Fig. 13A), whose levels are controlled also by the ubiquitin proteasome. The reduced ubiquitination of β-catenin resulted in increased levels of β-catenin in infected cells (Supplementary Fig. 13B) with a concomitant increase in the expression of β-catenin-controlled genes (Supplementary Fig. 13C). Control experiments showed that that expression of β*-catenin* was not increased by Kp52145 (Supplementary Fig. 13C).

The ubiquitination of proteins such as IκBα and β-catenin for their subsequent degradation by the ubiquitin proteasome is mediated by a common ubiquitin ligase, E3-SCF^β−TrCP^^55^. Therefore, we investigated whether *K. pneumoniae* may target E3-SCF^β-TrCP^. The covalent modification with NEDD8, termed neddylation, of the cullin-1 (Cul-1) subunit of the E3-SCF^β−TrCP^ is essential for the function of the ligase and, therefore, it is emerging as central regulatory event in cellular processes governed by the ubiquitin proteasome degradation of proteins^56^. Immunoblotting experiments revealed an overall decrease in the pattern of NEDD8-conjugated proteins in Kp52145-infected cells (Supplementary Fig. 13D). Moreover, Kp52145 induced the loss of Cul-1 neddylation (Fig. 6I). This was dependent on ROS and NLRX1 because Kp52145-indcued loss of Cul-1 neddylation was not observed in cells treated with the ROS inhibitor DPI or in *nlrx1* silenced cells (Supplementary Figs. 13E and Fig. 6J, respectively). As we anticipated, the neddylation of Cul-1 was not affected in cells infected with the *vgrG4* mutant (Fig. 6K); complementation of the mutant resulted in the loss of Cul-1 neddylation (Fig. 6K). Altogether, these findings support the notion that VgrG4 causes a loss in Cul-1 neddylation in a NLRX1-ROS-dependent manner, thereby affecting the function of E3-SCF^β-TrCP^.

We next questioned how NLRX1-ROS triggers the loss of Cul-1 neddylation. ROS signals can be transduced via the transient oxidative inactivation of catalytic cysteine residues present in certain regulatory enzymes^57^. Inspection of the enzymes of the neddylation machinery revealed the presence of putative redox-sensitive residues in Ubc12. This enzyme catalyses the transfer of a thioesterified NEDD8 moiety from its own active site to the Lys^720^ of cullin substrates^58^, making then Ubc12 a potential target of VgrG4-induced ROS to abolish Cul1- neddylation. Providing initial support to this hypothesis, studies using purified Ubc12 have demonstrated a NEDD8∼Ubc12 thioester form of Ubc12 is sensitive to oxidation^59^. We therefore evaluated the effects of *K. pneumoniae* on endogenous Ubc12. Immunoprecipitation experiments revealed the absence of Ubc12 modified with NEDD8 in Kp52145-infected cells (Fig. 6L). This was dependent on mtROS because the modification with NEDD8 was detected in infected cells pre-treated with mitoTEMPO (Fig. 6L). These data together with the published evidence indicate that VgrG4-induced NLRX1-mtROS abolishes the loading of Ubc12 with NEDD8 substrate.

Collectively, this evidence establishes that VgrG4-induced NLRX1-mtROS inhibits the activation of NF-κB. Mechanistically, NLRX1-ROS abolishes the neddylation of Cul-1 by oxidative inactivation of Ubc12, the NEDD8-conjugating enzyme, limiting the activity the E3-SCFβ-TrCP and thus the ubiquitination of IκBα, which, consequently, it is not degraded by the ubiquitin proteasome.

## Discussion

**Fig. 7:**
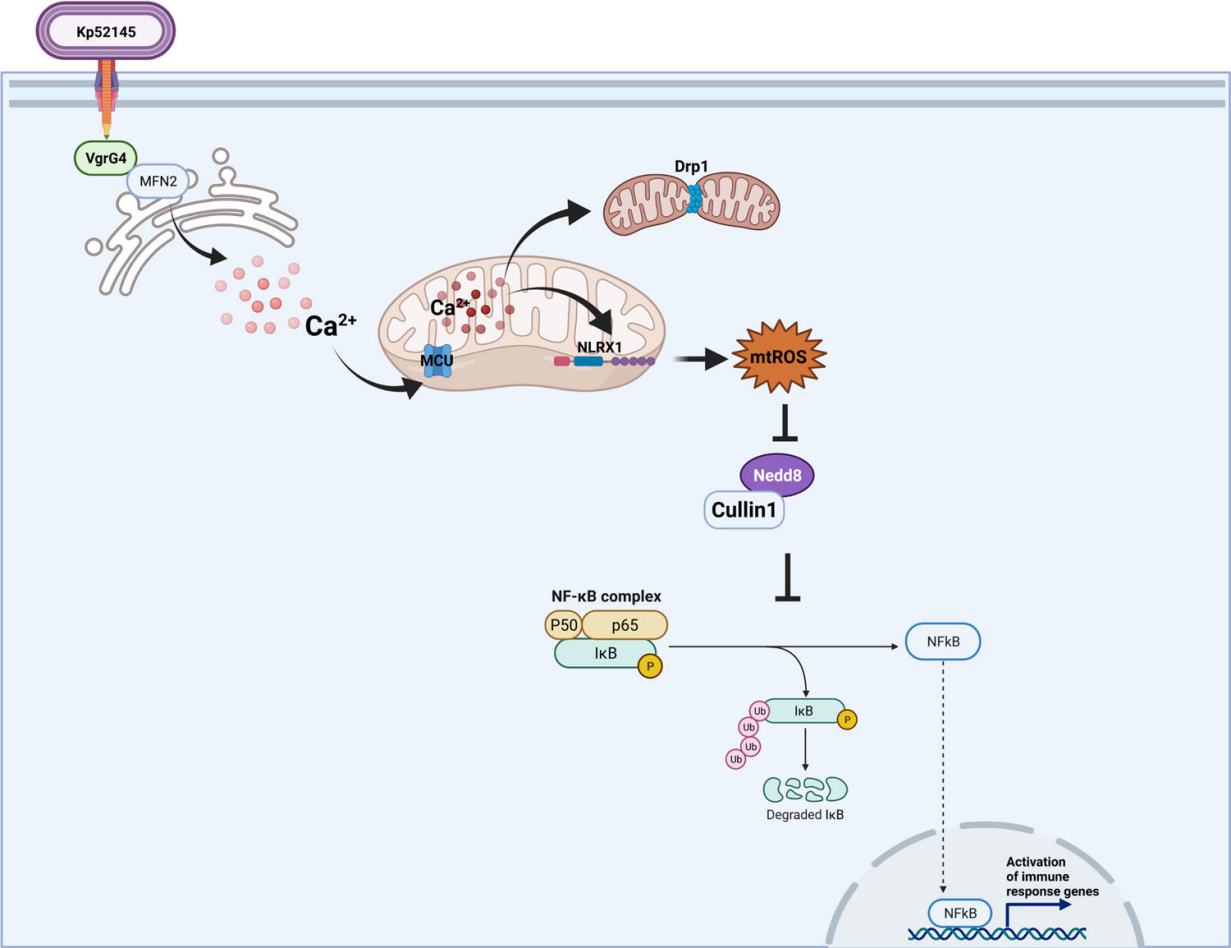
*K. pneumoniae* targets the mitochondria via its T6SS to promote infection. Working model of *K. pneumoniae* manipulation of the mitochondria in epithelial cells by exploiting its T6SS. Kp52145 injects the trans-kingdom T6SS effector VgrG4 into epithelial in a T6SS-dependent manner. VgrG4 colocalises with the ER-mitochondria tethering protein mitofusin 2 (MFN2). The ER localization of VgrG4 results in the influx of calcium from the ER to the mitochondria via the MCU channel. Mitochondria calcium induces the activation of Drp1 leading to the fragmentation of the mitochondria network. Additionally, mitochondria calcium activates the mitochondria innate receptor NLRX1 to produce ROS. NLRX1-elicted ROS abrogates the NEDDylation of Cul-1, essential for the function of the ubiquitin ligase, E3-SCF^β−TrCP^ responsible for the ubiquitination of the IκBα after its phosphorylation by the Iκκα/β kinase. The degradation of IκBα by the ubiquitin proteasome allows the nuclear translation of NF-κB to launch an antimicrobial programme. Therefore, by targeting the NEDDylation of Cul-1, VgrG4 subverts the activation of host defences. Created with BioRender.com.

Here, we establish that bacteria utilize the T6SS to target the mitochondria in yeast and mammalian cells. We demonstrate that *K. pneumoniae* exploits the trans-kingdom T6SS effector VgrG4 to fragment the mitochondria network in a Drp1-dependent manner (Fig. 7). Mechanistically, Drp1 activation results from VgrG4-triggered transfer of Ca^2+^ into the mitochondria from the ER (Fig. 7). Moreover, VgrG4-induced Ca^2+^ accumulation in the mitochondria also activates the innate receptor NLRX1 to produce mtROS. VgrG4-induced mtROS perturbs the ubiquitin ligase complex E3-SCF^β-TrCP^ to limit the activation of inflammatory responses governed by NF-κB (Fig. 7). Collectively, our findings unveil a hitherto unknown anti-eukaryotic function of the T6SS, expanding substantially the roles of this secretion system. Furthermore, this work illustrates a new anti-immunology axis exploited by a human pathogen to govern the activation of inflammatory responses.

*K. pneumoniae* exemplifies the global threat posed by antibiotic resistant bacteria. *K. pneumoniae*-triggered pulmonary infection has a high mortality rate reaching 50% even with antimicrobial therapy and may approach 100% for patients with alcoholism and diabetes. There is a wealth of knowledge on how *K. pneumoniae* develops resistance to different antibiotics^60^, however we still lack a complete understanding of what makes *K. pneumoniae* a successful pathogen. *Klebsiella* does not encode type III or IV secretion systems known to deliver effectors into immune cells, or any of the toxins affecting cell biology, making then interesting to identify the factor(s) utilized by the pathogen to manipulate cells. Our work revealed VgrG4 as a new type of anti-immunology weapon. However, we cannot rule that *Klebsiella* may inject additional T6SS effectors into cells. Future studies are warranted to elucidate whether *Klebsiella* exploits additional T6SS effectors to manipulate cells.

Compared to the number of antibacterial T6SS effectors, there are few anti-eukaryotic effectors characterized^9^. Additionally, they do not share any structural characteristic or target similar pathways, reflecting the versatility of the T6SS, facilitating a panoply of interactions with microbes and eukaryotic cells. VgrG4 showed a puncta distribution in epithelial cells, consistent with the aggregation of a number of VgrG4 monomers. This is in agreement with the biochemistry and structural data showing that VgrG proteins form at least trimeric structures^61^ that may contain even higher number of monomers inside the cell. Given the effect on mitochondria dynamics, it was somewhat unexpected to find out that VgrG4 did not colocalise with the mitochondria. Instead, our results demonstrate VgrG4 colocalises with the ER-mitochondria tethering proteins Mdm34 and Mfn2 in yeast and epithelial cells, respectively. Truncation experiments established that VgrG4 RTD containing domain is responsible for the interaction with Mfn2 whereas the VgrG domain mediates the interaction with Mdm34. The lack of structural homology between Mfn2 and Mdm34 explains why the same domain of VgrG4 cannot be responsible for the binding to both proteins. To the best of our knowledge, there are no reports of any T6SS effector showing ER colocalisation, and of any bacterial effector interacting with Mfn2.

The key molecular mechanism of VgrG4 action is the distribution of Ca^2+^ from the ER to the mitochondria. This Ca^2+^ accumulation activates Drp1, which is a critical regulator of mitochondrial fission within cells. Interestingly, Ca^2+^ is one of the signals modulating Drp1-mediated mitochondria fission in homeostasis^62^. Therefore, our results are consistent with the notion that VgrG4 affects a pathway governing mitochondria dynamics. Interestingly, Drp1 is emerging as target of bacterial pathogens as *Helicobacter pylori* and *Legionella pneumophila*-induced mitochondria fragmentation is Drp1-dependent^63,64^. However, this is not always the case because *Listeria monocytogenes*-induced fragmentation is Drp1-independent^65,66^.

The fragmentation of the mitochondria is not associated to a perturbation of the mitochondria physiology or cell death in stark contrast to what it is observed in other pathogens^63–65^. The only phenotype we found is the release of mtDNA to the cytosol that activates STING signalling to produce type I IFN and ISGs. We have recently uncovered the importance of type I IFN signalling in host defence against *K. pneumoniae*^67^. Nonetheless, the possibility exists that other STING controlled responses may be beneficial for the pathogen survival. Future studies are warranted to characterize the role of STING signalling in *K. pneumoniae* infection biology.

The accumulation of Ca^2+^ in the mitochondria also results in the activation of NLRX1 to produce mtROS. NLRX1 remains an enigmatic innate receptor. Only recently, the localisation of NLRX1 within the matrix of the mitochondria has been conclusively established^49^. There is conflicting data concerning NLRX1 function as a negative or positive regulator of inflammation^68–71^. Moreover, the ligand or activating signal of NLRX1 upon infection remains unknown. There is only consensus on its role in ROS production following infection^69,72^. Our evidence reveals a role for NLRX1 limiting inflammation following *K. pneumoniae* infection. Our results establish that the accumulation of Ca^2+^ in the mitochondria is sufficient to induce NLRX1-controlled mtROS. These findings lead us to propose that NLRX1 has evolved to detect the action of pathogens on mitochondria using the non-homeostatic accumulation of Ca^2+^as a signal. Future studies are warranted to confirm this hypothesis following the infection with other bacteria and viruses.

Another novel finding of this work is that VgrG4-induced activation of NLRX1 decreases the NEDDylation of Cul-1 in a ROS-dependent manner to limit the activation of NF-κB. There are no reports of T6SS effectors affecting the posttranslational modification of proteins, being VgrG4 one of the few bacterial effectors doing so^73^. The NEDDylation of Cul-1 is essential for the function of the ubiquitin ligase complex E3-SCF^β-TrCP^ that controls the levels of proteins involved in host-pathogen interactions, making Cul-1 an attractive target to control cell biology. Further suggesting that Cul-1 is a prime *Klebsiella* target, we have recently demonstrated that *K. pneumoniae* induces the expression of the deneddylase CSN5 to limit the modification of Cul-1 with NEDD8^74^. Targeting the ubiquitin ligase complex E3-SCF^β-TrCP^ via Cul-1 is an efficient strategy to interfere simultaneously with a number of signalling pathways instead of targeting directly key proteins of each pathway as it has been described for pathogens such *as Listeria, Salmonella, Legionella, E. coli* or *Shigella*. This may explain the need for these pathogens to encode a portfolio of effectors to manipulate cell biology, being the extreme case *Legionella* encoding more than 300 effectors. In this work, we have only focused on the NF-κB signalling cascade given its crucial importance in defence against *K. pneumoniae* infections^25,51–53^. However, we hypothesize a number of other pathways modulated by E3-SCF^β-TrCP^ will be out of balanced in VgrG4-intoxicated cells. Providing initial support, we have shown the β-catenin pathway, whose activation is dependent on the E3-SCF^β-TrCP^-controlled levels of β-catenin, is upregulated in *Klebsiella*-infected cells.

The manipulation of mitochondria to subvert inflammation via NLRX1 activation in a ROS-dependent manner is an underappreciated anti-immunology strategy. Previous work from our laboratory demonstrated that *Klebsiella* also exploits two other innate receptors, NOD1, and TLR4, to control cell responses^52,53,74^. Therefore, the theme taking shape is that a signature of *K. pneumoniae* infection biology is to hijack the activation of innate immune receptors to counteract the activation of host antimicrobial pathways. This strategy is radically different to those employed by other pathogens such *as Listeria, Salmonella, Legionella* and *Shigella*, who deploy bacterial proteins to prevent the activation of signalling upon sensing the infection.

Host-directed therapeutics aiming to interfere with host factors required by pathogens to counter the immune system are emerging as untapped opportunities that are urgently needed in the face of the global pandemic of antibiotic resistant infections. Our results suggest that limiting *Klebsiella*-governed mitochondria manipulation should affect decisively the host-*Klebsiella* interface contributing to the clearance of the infection. Mitochondrial dysfunction also contributes to the pathology of many common disorders, including neurodegeneration, metabolic disease, heart failure, and ischaemia–reperfusion injury. There are efforts to develop pharmacological approaches aimed at therapeutically restoring mitochondrial function, and a number of agents have entered clinical trials^75^. We propose that these drugs shall show a beneficial effect to treat *K. pneumoniae* infections alone or as a synergistic add-on to antibiotic treatment. Future studies shall confirm whether this is the case.

## Methods

### *Saccharomyces cerevisiae* strains and growth conditions

The *S. cerevisiae* strains used in this study were YPH499 (*MAT*a *ura3-52 lys2-801_amber ade2-101_ochre trp1-Δ63 his3-Δ200 leu2-Δ1)*, diploid SLY001 for general purposes, and VHY87 (*MATα leu2-3, 112 ura3-52 his4 can*^*R*^ *TRP1::DsRed-HDEL*), a gift of M. Cyert (Stanford University, CA, USA), for the visualization of the ER. The SLY001 diploid strain was obtained by crossing strains of opposite mating type BY4741 *trp1Δ* (*MAT*α *his3Δ1 leu2Δ0 met15Δ0 ura3Δ0 trp1Δ::kanMX4*) and BY4742 *trp1Δ* (*MAT*a *his3Δ1 leu2Δ0 met15Δ0 trp1Δ ura3Δ0*) (Supplementary Table 1). Strains deleted for *DNM1* and *FIS1* were obtained from the yeast whole genome deletion (WGD) collection (Euroscarf) on the BY4741 background. Plasmid YEplac112-Ilv6-mCherry for the visualization of mitochondria was previously described^76^.

YPD (1% yeast extract, 2% peptone and 2% glucose) broth or agar was the non-selective medium used for growing yeast. For plasmid selection and maintenance was used selective synthetic dextrose media (SD) (0.17% yeast nitrogen base without amino acids, 0.5% ammonium sulphate, 2% glucose, 0.12% drop-out supplements mixture and was supplement with the appropriate amino acids and nucleic acid bases). SR and SG media were SD with 1.5% (w/v) raffinose or 2% (w/v) galactose instead of glucose. All galactose induction experiments were performed by growing transformants in SR liquid medium for 18 h at 30 °C to log-phase and then suspended in SG medium during 4-6 hours at 30 °C.

### Molecular techniques and construction of yeast expression plasmids

Transformation of yeast by the lithium acetate and *E. coli* associated molecular techniques were performed by standard procedures.

Invitrogen Gateway Cloning (Thermofisher) was used to fuse the hemagglutinin (HA) tag protein or red fluorescent protein (DsRed) to ERMES proteins Mdm34 and Mmm1. *MDM34* and *MMM1* entry clones in pDONR221 (a gift from Elba del Val and Julia María Coronas-Serna) were subcloned into a pAG413GPD-ccdB-HA or pAG424-GAL-ccdB-DsRed destination vectors by LR recombination reaction (Addgene kit #1000000011) to obtain the expression clone pAG413-GPD-Mdm34-HA, or the plasmids pAG424-GAL-Mdm34-DsRed and pAG424-GAL-Mmm1-DsRed. pEG(KG)-VgrG4 expressing GST fusions of VgrG4 and truncated versions were described before^12^. pEG(KG)-GST-VgrG4∆RTD was constructed by overlap extension PCR, amplifying separately the *vgrG4* N-terminal-coding part and the one encoding the 837-899 extension. The fused amplicon bearing *XbaI* sites introduced in the 5’ region of the primers was cloned into the *XbaI*-cleaved pEG(KG) expression vector in frame with GST. Primers used for amplifying the N-terminal extension are UP-1-157XbaI and LO 1-517overlap838. The ones used for amplifying the 837-899 extension are UP-838overlap517 and LO-838-899XbaI (listed in Supplementary Table 2). pYES2-GFP-VgrG4 and pYES2-GFP-VgrG4(518-C) were constructed by PCR amplification of *vgrG4* or the region starting in codon 518, respectively, by using primers bearing *Bgl*II sites in 5’ and ligating the amplicons into *Bam*HI-cleaved pYES2-GFP^77^ Primers used were UP-VgrG4-FL or UP-VgrG4-518, respectively, and LO-VgrG4 (Supplementary Table 2). pYES2-GFP-VgrG4(1-517) was constructed by substituting the codon 518 in the *vgrG4* sequence by a stop codon using *Dpn*I-based site directed mutagenesis with the pYES2-GFP-VgrG4 as a template and primers (1-517)−1 and (1-517) (Supplementary Table 2).

### Evaluation of oxidative damage in yeast cells

ROS generation was tested by flow cytometry. Cells were cultured in SR medium lacking uracil and leucine overnight. Then, cultures were suspended in SG medium during 16 hours at 30 °C. As a positive control, oxidative damage was induced by adding 125 mM acetic acid during 4 hours after 12 hours of galactose induction at one of the control samples. After that, 1 mL of each culture was treated with 2,5 μg/mL of dihydroethidium during 5 minutes at 30 °C under stirring. The samples were diluted 1:10 in PBS. Cells were analysed using a 419 FACScan (Becton Dickinson) flow cytometer through a 488 nm excitation laser and a 585/42 BP emission filter (FL2). At least 10,000 cells were analyzed for each experiment. Data was processed using FlowJo software (FlowJo LLC, Ashland, OR, USA).

### Microscopy in yeast cells

For fluorescence microscopy to visualise GFP- and DsRed-expressing cells, transformants were cultured in SR medium for 18 hours at 30 °C, then, cells were suspended into SG medium and incubated for 4-6 hours for *GAL1* promoter induction. Finally, cells were harvested by centrifugation and observed directly. Cells were examined under Eclipse TE2000U microscope (Nikon, Tokyo, Japan) and digital images were acquired with an Orca C4742-95-12ER charge-coupled-device camera (Hamamatsu Photonics, Hamamatsu City, Japan) and processed by HC Image (Hamamatsu).

### GST pull-down in yeast cells

Yeast cell cultures were grown in SR medium lacking uracil, leucine and histidine for 18 hours at 30 °C, then the appropriate number of cells was suspended in 100 mL of SG medium for 5-6 h to reach exponential phase and *GAL1* promoter induction. Standard procedures were used for collection and breakage. Cells were spun and lysed in 300 µL of ice-cold lysis buffer [10% v/v glycerol, 50 mM Tris/HCl pH 7.5, 0.1% NP40, 150 mM NaCl, 5 mM EDTA pH 8, 50 mM NaF, 5 mM sodium pyrophosphate, 50 mM beta-glycerol phosphate, 1 mM sodium vanadate supplemented with PMSF up to 1 mM and protease inhibitor cocktail (Roche/Sigma-Aldrich)]. Then, the extracts were diluted if necessary to balance protein concentration, and 10 µL were preserved as “inputs” at −80 °C overnight. To equilibrate the beads, three washes were performed with lysis buffer, and then 50 µL of glutathione sepharose 4B beads (GE Healthcare Life Sciences) diluted at 50% were added to 200 µL of each extract. After an overnight incubation with the beads at 4 °C, samples were centrifuged, the supernatant was discarded, and beads were washed 6 times with lysis buffer. Finally, they were suspended in 25 µL of SDS‐PAGE loading buffer (1 M Tris/HCl pH 6.8, 10% SDS, glycerol, 1 M DTT and bromophenol blue). 10 µL of the original lysates were preserved as “inputs” at -80 °C overnight and then suspended in 10 µL of the same SDS-PAGE loading buffer. Cells were boiled at 99 °C during 5 min. In the gels, 10 µL of each input and 20 µL of the samples after affinity purification were loaded. Standard procedures were used for protein separation by SDS-PAGE, and transfer to nitrocellulose membranes.

Anti-HA antibody (Anti-HA High Affinity, Rat monoclonal antibody (clone 3F10), Sigma-Aldrich) diluted 1:1000 was used to detect both HA-tagged ERMES proteins Mdm34. GST fusion proteins were detected with anti-GST antibody (Anti-GST rabbit polyclonal Antibody (Z-5): sc-459 (Santa Cruz Biotechnology) diluted 1:5000. Primary antibodies were detected using IRDye800 anti-rat (Li-Cor Biosciences) or Alexa-680 anti-mouse (Invitrogen) antibodies with an Odyssey Infrared Imaging System (Li-Cor Biosciences).

### Cell culture

Lung epithelial A549 cells (ATCC CCL-185) were grown in Roswell Park Memorial Institute (RPMI) 1640 Medium (Gibco 21875) supplemented with 10% heat-inactivated fetal bovine serum (FBS), 10 mM HEPES (Sigma), 100 U mL-1 penicillin, and 0.1 mg mL-1 streptomycin (Gibco) at 37 °C in a humidified 5% CO2 incubator. Human airway epithelial cells NuLi-1 (ATCC CRL-4011) were grown in PromoCell Small Airway Epithelial Cell Growth Medium (Promocell, C-21070) at 37 °C in a humidified 5% CO2 incubator. Flasks and plates were coated with collagen type IV from placenta (Sigma C-7521). Cells were routinely tested for *Mycoplasma* contamination.

### Bacterial strains and infection conditions

Bacterial strains and plasmids used in this study are shown in Supplementary Table 2. Kp52145 is a clinical isolate, serotype O1:K2, belonging to the virulent CC65 clonal complex. When appropriate media were supplemented with antibiotics at the following concentrations: chloramphenicol (Cm) 25 μg/mL, carbenicillin (Cb) 50 μg/mL, spectinomycin (spec) 50 μg/mL.

*K. pneumoniae* strains were grown in 5 mL LB at 37 °C, harvested at mid exponential phase (2500 × *g*, 20 min) and adjusted to an optical density of 1.0 at 600 nm in PBS (5 × 10^8^ CFU/mL). All the strains containing a pBAD30 plasmid were induced for 30 min with 0.05% (v/v) of arabinose prior to harvesting. *Klebsiella* infections were performed using a multiplicity of infection (MOI) of 100 bacteria per cell.

*Y. enterocolitica* strains were grown overnight in 5 mL LB at 21 °C, refreshed 1:10 (v/v) in Tryptic Soy Broth (TSB) media supplemented with 20 mM magnesium chloride and 20 mM sodium oxalate for 3h30 at 21 °C followed by 30 min at 37 °C. Infections were performed using a MOI of 50 bacteria per cell and media was replaced at 30 minutes post infection for media containing gentamicin (100 µg/mL) to kill any extracellular bacteria.

Infections were performed in the same media, without antibiotics, used to maintain the cell line and incubated at 37 °C in a humidified 5% CO2 incubator. Infections of A549 epithelial cells were performed two days after seeding including a serum starvation step 16 h before infection using RPMI 1640 Medium (Gibco 21875) supplemented only with 10 mM HEPES. Infections of NuLi-1 cells were performed two days after seeding.

### Construction of VgrG4 tagged with GSK3β

To allow the study of vgrG4 translocation by Kp52145 into epithelial cells, the *vgrG4* gene was fused with a GSK3β tag that can only be phosphorylated by mammalian cells. The tag was generated by hybridisation of the primers GSK3b_F1 and GSK3b_R1 (Supplementary Table 2). The product of this PCR was cloned into *Sma*I site of pBAD30 and transformed into *E*. *coli* β2163, and then mobilized into *K. pneumoniae* by conjugation.

### Construction of a *Y. enterocolitica* strain delivering VgrG4 in a T3SS-dependent manner

To allow the study of *vgrG4* translocation into cells we used a *Y. enterocolitica* strain that delivers VgrG4 via the Ysc T3SS because *vgrG4* is fused to the N-terminal secretion/translocation domain of YopE (the first 53 amino acids of this protein)^32^. This strain carries a mini-virulence plasmid (pT3SS), encoding the genes for a functional T3SS, the gene for the Yersinia adhesin (YadA) and a second plasmid containing the T3SS substrate YopE and the cognate chaperone SycE^32^. This strains does not encode any other T3SS effector^32^. The coding region of *vgrG4* was amplified using primers Kpn52_*vgrG4*_VSVG_F1 and Kpn52_*vgrG4*_VSVG_R1 and digested with *Bgl*II and *Xho*I. Fragments were gel purified and subcloned into the vector pYOPE53 previously digested with *Bam*HI and *Sal*I. The plasmid was transformed into *E*. *coli* β2163, and then mobilized into *Y. enterocolitica* by conjugation. VgrG4 truncated versions were generated using the primers Kpn52_VgrG4_VSVG_F1 and Kpn52_VgrG4_VSVG_R2 for the N-terminal version (1-517) or Kpn52_VgrG4_518-C and Kpn52_VgrG4_VSVG_R1 for the C-terminal (518-C). Primer sequences are listed in Supplementary table 1.

### Transfection conditions

Transfection of A549 cells with siRNAs or plasmids was carried out using Lipofectamine 2000 (Invitrogen) lipofection reagent according to manufacturer’s instructions. For transfection of siRNAs, 1.2 × 10^5^ cells (12-well plate) were transfected in suspension with 20 nM siRNA using 2 μL of Lipofectamine 2000 in a final volume of 1 mL. For transfection of the MFN2-YFP plasmid, 3.0 × 10^5^ cells (6-well plate for immunoprecipitation) or 5.0 × 10^4^ cells (24-well plate for microscopy) were transfected the day after seeding with 2 µg (6-well) or 0.5 µg (24-well) using 6 μL or 1 µL of Lipofectamine 2000 in a final volume of 2 mL or 0.5 mL, respectively. MFN2-YFP was a gift from Richard Youle (Addgene plasmid # 28010^78^). Infections were carried 48 h post transfection.

All siRNA duplexes used for in vitro studies were chemically synthesised by Dharmacon (Horizon Discovery Group). The following siRNAs sense sequences were used: hMCU (ON-TARGETplus Human MCU, Dharmacon, J-015519-18), hDrp1 (5′-ACAAGUCGGCGGUGGAGUA-3′), hNLRX1 (ON-TARGETplus Human NLRX1 SMARTPool, Dharmacon, L-012926-01), hVAPB (5’-UGUUACAGCCUUUCGAUUAdTdT-3’), hMFN2 (5’- GACUAUAAGCUGCGAAUUAdTdT-3’). An AllStars (AS) Negative Control scrambled siRNA (Qiagen) with no homology to any known mammalian gene was used as a negative control. Efficiency of transfection was confirmed by qPCR analysis of duplicate samples from three independent transfections by normalising to the glyceraldehyde 3-phosphate dehydrogenase (h*GAPDH*) gene and comparing gene expression in the knockdown sample with the luciferase negative control. Primers used are listed in Supplementary Table 1. The percentage of transcript remaining after transfection is presented in (Supplementary Fig. 2).

### Immunoblot analysis

A549 were seeded in twelve-well plates (1.2 × 10^5^ cells per well) and grown for 48 h prior to infection. Cells were infected with *K. pneumoniae* or *Y. enterocolitica* strains for different time points as indicated in the figure legends. Cells were then washed in 1 mL of ice-cold PBS and lysed in 80 μL of 2 x SDS sample buffer (1x SDS Sample Buffer, 62.5 mM Tris-HCl pH 6.8, 2% w/v SDS, 10% glycerol, 50 mM DTT, 0.01% w/v bromophenol blue). The cell lysates were sonicated for 10 seconds at 10% amplitude (Branson Sonifier), boiled at 95 °C for 5 minutes and centrifuged at 12,000 *×* *g* for 1 min. 20% of the cell lysates were resolved by standard 8 or 12% SDS-PAGE and electroblotted onto nitrocellulose membranes. Membranes were blocked with 4% bovine serum albumine (w/v) in TBST and protein bands were detected with specific antibodies using chemiluminescence reagents and a G:BOX Chemi XRQ chemiluminescence imager (Syngene).

The following antibodies were used: anti-VSVG (1:1000, Sigma V4888), anti-phospho-GSK3β (1:1000, Cell Signaling Technology (CST) 5558), anti-GSK3β tag (1:1000, CST 9325), anti-GSK3β (1:1000, Santa Cruz Biotechnology sc-67075), anti-phospho-DRP1 (Ser616) (1:1000, CST 4494), anti Drp1 (1:1000 CST 8570), anti-Cullin-1 (1:200, Santa Cruz Biotechnology sc-12761), anti-NEDD8 (1:1000, Invitrogen 34-1400), anti-Ubc12 (1:1000, Santa Cruz Biotechnology sc-366017), anti-β-catenin (1:1000, Santa Cruz Biotechnology sc-7199), anti-K48 Polyubiquitin (1:1000, CST 4289), anti-IκBα (1:1000, CST 4814), anti-phospho-Iκκαβ (1:1000, CST 2697), anti-phospho-IκBα (1:1000, Santa Cruz Biotechnology sc-7977), anti-GFP (1:5000, Proteintech 66002), anti-Tom20 (1:500, Santa Cruz Biotechnology F-10 sc-17764), anti-calnexin (1:1000, Santa Cruz Biotechnology AF-18 sc-23954), anti-E.coli RNA polymerase α (1:5000, BioLegend 663102).

Immunoreactive bands were visualized by incubation with horseradish peroxidase-conjugated goat anti-rabbit immunoglobulins (1:5000, BioRad 170-6515), goat anti-mouse immunoglobulins (1:5000, BioRad 170-6516) or mouse anti-goat immunoglobulins (1:5000, Santa Cruz Biotechnology sc-2354).

To detect multiple proteins, membranes were reprobed after stripping of previously used antibodies using a pH 2.2 glycine-HCl/SDS buffer. To ensure that equal amounts of proteins were loaded, blots were reprobed with mouse anti-human tubulin (1:3000, Sigma T5168).

### Densitometry analysis

Drp1 blots were quantified using Image Studio Lite version 5.2 (Li-cor) and normalized to the loading control. Graphs represent fold change compared to non-infected cells set to 100.

### Co-immunoprecipitation analysis

Cells were seeded (3 × 10^5^ cells per well) in six-well plates and two wells were used per sample. At the designated time points, cells were washed with pre-chilled PBS (1 mL) and then lysed with 500 μL of pre-chilled RIPA buffer (1% [w/v] Triton X-100, 1% [w/v] Sodium Deoxycholate, 0.1% [w/v] SDS, 0.15 M NaCl, 50 mM Tris-HCl, pH 7.2, 1 mM PMSF and Halt Protease Inhibitor Cocktail (Thermo Scientific)) for 30 min on ice. Lysates were centrifuged at 12,000 *×* *g* for 15 min at 4 °C. Supernatants were transferred to pre-chilled tubes and incubated for 2 h with the appropriate antibody (1 μg) at 4 °C with rocking. Normal mouse IgG (Santa Cruz Biotechnology, sc-2025) was used as the antibody binding control. This was followed by the addition of protein A/G agarose beads (20 μL per sample; Santa Cruz Biotechnology, sc-2003) and incubation overnight at 4 °C with rocking. Immunoprecipitates were collected by centrifugation at 1000 *×* *g* for 2 min at 4 °C and the beads were then washed two times with RIPA buffer (500 μL) lacking the protease inhibitor mixture. The beads were resuspended in 2x sample buffer (40 μL) and boiled for 5 min at 95 °C. Samples were centrifuged at 12,000 × *g* for 1 min and subjected to immunoblotting. The immunoreactive bands were detected using HRP conjugated TidyBlot (BioRad, 1:1000).

### Cell fractionation

Epithelial cells were seeded in a 10 cm dish, at a cell density of 1 × 10^6^ cells per dish, and two dishes were used per sample. Cells were infected as described previously for 90 min with *Y. enterocolitica* strains. To isolate mitochondria from endoplasmic reticulum fractions a protocol was adapted from Wieckowski and colleagues^79^. Briefly, cells were washed with PBS, scrapped and homogenised in 2 mL of IB buffer [225 mM mannitol, 75 mM sucrose, 0.1 mM EDTA and 30 mM Tris-HCl (pH 7.4)] using a pestle. Lysates were centrifuged at 600 × *g* for 5 min at 4 °C. Supernatants were transferred to pre-chilled tubes and centrifuged at 7000 × *g* for 10 min at 4 °C. The resulting pellets were resuspended in 1 mL of buffer IB without EDTA and centrifuged at 7000 × *g* for 10 min at 4 °C (resulting pellet is the mitochondrial fraction). The supernatants were centrifuged at 20,000 × *g* for 60 min at 4 °C to isolate the ER fraction (resulting pellet).

### Fluorescence dye-based detection of ROS

Epithelial cells were seeded in a 96-well black clear bottom plate, at a cell density of 2 × 10^4^ cells per well. Cells were infected as described previously for 5 h with *K. pneumoniae* strains or for 90 min with *Y. enterocolitica* strains. Cells were treated with 50 µM 2′,7′-Dichlorofluorescein (Sigma, 35848) for 30 min, washed twice with pre-warmed Hanks’ Balanced Salt Solution (HBSS) and fluorescence was measured in a POLARStar Omega BMG LabTech plate reader at Excitation/Emission of 485/520 nm. All the experiments were carried out in triplicate wells in three independent occasions.

### ATP determination

A549 cells were seeded in 12 well plates at a density of 1 × 10^5^/well two days prior to infection. Cells were starved for 16 h using RPMI 1640 Medium supplemented only with 10 mM HEPES and changed to media without antibiotics prior to infection. Cells were infected with *K. pneumoniae* for 5 h, and with *Y. enterocolitica* strains for 2 h. Cells were lysed in 0.5% (w/v) saponin in PBS and 20% of the cell lysate was used to determine ATP concentration using an ATP Determination Kit (Invitrogen, A22066) following manufacturer’s instructions and measured in a Promega Glomax with an integration time of 0.1 seconds.

### Mitochondrial membrane potential determination

Epithelial cells were seeded in a 96-well black clear bottom plate, at a cell density of 2 × 10^4^ cells per well. Cells were infected as described previously for 3 h with *K. pneumoniae* strains or 1h30 with *Y. enterocolitica* strains. Mitochondrial Membrane Potential was assessed with a TMRM assay kit (Abcam, ab228569) following manufacturer’s instructions. Briefly TMRM was added to the culture media and incubated for 30 minutes after which cells were washed in 0.2% (w/v) BSA in PBS, bathed in Live Cell Imaging Buffer and fluorescence was measured in a POLARStar Omega BMG LabTech plate reader at an Excitation/Emission of 544/590 nm.

### Seahorse analysis

A549 cells were seeded in Seahorse XF Cell Culture Microplates at a density of 2 × 10^4^ cells per well two days prior to infection. Cells were starved for 16 h using RPMI 1640 Medium supplemented only with 10 mM HEPES and changed to media without antibiotics prior to infection. Seahorse cartridge was hydrated overnight in Seahorse XF Calibrant solution at 37 °C overnight in a non-CO_2_ incubator. Infection was performed at a 100:1 M.O.I. and after 3 h contact, cells were washed and media replaced with XF culture media (pH 7.4 supplemented with 1 mM pyruvate, 2 mM glutamine, 10 mM glucose and 30 µg/mL gentamicin to remove extracellular bacteria). Metabolic activity of cells was assessed using the Seahorse Mito Stress Test Kit (Ref: 103015-100, Agilent) according to manufacturer’s instructions and experiment analysed with the Seahorse XFe96 analyser. 8 wells were used per condition across 3 independent experiments.

### Determination of cytosolic mitochondrial DNA

A549 or NuLi-1 cells were grown as monolayers in 10 cm dishes (1.0 × 10^6^ cells), infected with *Y. enterocolitica* strains for 2 hours after which cells were washed in ice-cold PBS, scraped from culture dishes on ice using a plastic cell scraper and collected in micro-centrifuge tubes in 1 mL of ice-cold PBS. After centrifugation at maximum speed for 10 seconds, cell pellets were resuspended in 900 μL of ice-cold 0.1% NP40 in PBS to lyse the cells. One third of this cytosolic fraction was then submitted to a PCR clean up kit (Qiagen) and the resulting DNA was submitted to qPCR for mitochondrial cytochrome oxidase 1 (mtCO1) using as housekeeping gene human DNA polymerase β (Polβ).

### Mitochondria calcium detection

Epithelial cells were seeded in a 96-well black clear bottom plate, at a cell density of 2 × 10^4^ cells per well two days prior to infection. Cells were starved for 16 h using RPMI 1640 Medium supplemented only with 10 mM HEPES and changed to media without antibiotics prior to infection. Cells were infected for 30 minutes with *Y. enterocolitica* strains after which media was replaced with HBSS supplemented with 100 µg/mL gentamicin to remove any extracellular bacteria and with 50 µM Rhod2-AM (Sigma, catalogue number 35848). Fluorescence was measured in a POLARStar Omega BMG LabTech plate reader at an Excitation/Emission of 544/590 nm at 20 minutes interval for 2 hours. All the experiments were carried out in triplicate wells in three independent occasions.

### Mitochondrial ROS detection

Epithelial cells were seeded in a 96-well black clear bottom plate, at a cell density of 2 × 10^4^ cells per well. Cells were treated with 50 µM MitoSOX Red Mitochondrial Superoxide Indicator (Invitrogen, M36008) for 30 minutes and were replenished with fresh antibiotic free media. Cells were then infected as described previously for 5 h with *K. pneumoniae* strains or for 1h30 with *Y. enterocolitica* strains. After infection cells were washed twice with pre-warmed Hanks’ Balanced Salt Solution (HBSS) and fluorescence was measured in a POLARStar Omega BMG LabTech plate reader at Excitation/Emission of 544/590 nm. All the experiments were carried out in triplicate wells in three independent occasions.

### Cell viability

The thiazolyl blue tetrazolium bromide (MTT) assay was used to determine cell viability. Cells were seeded at a concentration of 1 × 10^5^ cells/mL in 96-well plates and infected under normal conditions. After 3 h, 10 μL of MTT (5 mg/mL in PBS) was added and the plates incubated at 37 ^o^C in a humidified incubator for 3 hours. Media was removed from all wells and replaced with 100 µL DMSO to dissolve any dye crystals, and incubated for 30 minutes at 37 ^o^C in a humidified incubator. Staining was then quantified by determining the OD_570_ in a plate reader.

### Generation of the A549 mCherryER cell line

To generate a stable cell line with fluorescently-labelled ER, the *mCherry* gene including KDEL ER-targeting sequence and Calreticulin ER-retention signal was amplified by PCR (5’-CTAGGGATCCGCC ACCATGCTGCTATCCG-3’; 5’-TCTACTCGAGTTACAGCTCATCCTTCTTGTACAGCTCGTCCATGC-3’) from pmCherryER/ pICC1584^80^ and inserted into the retroviral expression vector pMXs-IRES-Puro using standard restriction enzyme (*Bam*HI, *Xho*I) cloning. Attenuated retrovirus for transduction was produced as described previously (PMID: 26216420). Briefly, Hek293E cells were transfected with pMXs-IP and the pCMV-VSV-G envelope and pCMV-MMLV-gag-pol packaging plasmids, the medium replaced with fresh growth medium after 24 h and cell supernatant containing virions harvested after another 24 h of incubation. A549 cells were infected for 24 h, washed and growth medium containing 1.5 µg/mL puromycin added. After 7 days of selection including media exchange to remove dead cells and visual confirmation of mCherry fluorescence throughout the culture, the attached cells were harvested, and stocks frozen and used for experiments without further selection.

### Microscopy

A549 or NuLi-1 cells were seeded (5 × 10^4^ cells per well) in 24-well plates with sterile round glass cover slips, grown for 24 h to 70-80% confluence and starved for 18 h. Cells were pre-incubated with 50 µM Mitotracker Red FM (Invitrogen, M22425) for 30 minutes and media was replaced to antibiotic free medium prior to infection. After incubation with either bacterial strains or PBS (control) for the indicated time points in figure legends, medium was removed, and the cells were gently washed twice with PBS. Cells were then fixed by the addition of 4% (w/v) paraformaldehyde for 20 min. Cells were washed twice with PBS and kept at 4 °C in PBS supplemented with 1 mM NH_4_Cl until staining. Nuclei were stained with Hoechst 33342 (1.5 μg/mL; Sigma) for 30 minutes, washed with PBS and were mounted with ProLong Gold Antifade Mountant (Molecular Probes Inc). Fluorescence images were captured using the ×100 objective lens on a Leica SP8 laser scanning confocal microscope equipped with the appropriate filter sets. Acquired images were analysed using the LAS imaging software (version 3.7, Leica). Quantitative analysis of mitochondrial morphology was performed using the plugin for ImageJ Mitochondria Analyzer in 20 images for each condition from at least three independent experiments (https://github.com/AhsenChaudhry/Mitochondria-Analyzer)^31^.

Analysis of VSV-G co-localisation was performed using anti-VSVG (1:400, Sigma V4888) or anti-Flag (in the case of Kp52145 co-localisation) (1:5000, Sigma F3165) stained for 1 hour followed by Alexa Fluor 647 (1:400, Life-tech A21244), Alexa Fluor 488 (1:400, Life-tech A21206) or Alexa Fluor 488 (1:400, Abcam ab150117).

Analysis of p65 translocation was determined in cells fixed as above and stained with p65 antibody (1:400, Santa Cruz Biotechnology sc-372) using Alexa Fluor 488 (1:400, Life-tech A21206) with fluorescence images acquired in a Leica DM5500 microscope using the ×64 objective lens.

### Transmission electron microscope

A549 cells were seeded (3 × 10^5^ cells per well) in 6-well plates, grown for 24 h to 70–80% confluence and starved for 18 h. Media was replaced to antibiotic free medium prior to infection and after incubation with either Kp52145 or PBS (non-infected control) for 3 hours, medium was removed, and the cells were gently washed twice with PBS. Cells were then fixed by the addition of 2.5% (v/v) glutaraldehyde + 1.5% (v/v) paraformaldehyde in PHEM buffer (Electron Microscopy Sciences, catalogue number 11165) for 1 hour at room temperature. Cells were them scraped and collected into a microcentrifuge tube. Cell suspension was pelleted at 800 *g* for 15 minutes and embedded in agarose (2%, w/v, in PHEM buffer). Cells were then post fixed with 2% osmium tetroxide (v/v in PHEM) and immersed in Spurr replacement (Low viscosity resin kit, TAAB, T262) and polymerised at 60 °C for 48 hours. The block was trimmed, thin sectioned (90–100 nm thick) and then applied to copper grids and air dried. The grid was loaded in a JEOL1400 transmission electron microscopy (Jeol). At least 10 randomly selected electron micrographs from each sample were examined.

### RNA extraction and quantitative real‐time PCR analysis

Cells were washed twice in PBS and RNA was extracted using TRIzol Reagent (Ambion) according to the manufacturer’s instructions. Duplicate cDNA preparations from each sample were generated from 1 μg of RNA using Moloney murine leukaemia virus (M‐MLV) reverse transcriptase (Sigma‐Aldrich) according to the manufacturer’s instructions. Quantitative real‐time PCR analysis of gene expression was undertaken using the KAPA SYBR FAST qPCR Kit and a Rotor-Gene Q real-time PCR cycler System (Qiagen). Thermal cycling conditions were as follows: 95 °C for 3 min for enzyme activation, 40 cycles of denaturation at 95 °C for 10 s and annealing at 60 °C for 20 s. Primers used in qPCR reactions are listed in Supplementary Table 1. cDNA samples were tested in duplicate and relative mRNA quantity was determined by the comparative threshold cycle (ΔΔC_T_) method, using glyceraldehyde 3-phosphate dehydrogenase (h*GAPDH*) gene normalisation.

### Quantification of cytokines

Infections were performed in twelve-well plates (1.2 × 10^5^ cells per well) using Kp52145 at a MOI of 100:1. After 3 hours of infection media was replaced by media containing gentamicin to clear any extracellular bacteria and supernatants from infected cells were collected after 16 h and spun down at 12,000 × *g* for 5 min to remove any debris. IL-8 in the supernatants was determined using a Human IL-8 Standard TMB ELISA Development Kit (PeproTech, catalogue number 900-T18), according to manufacturer’s instructions. Experiments were run in duplicate and repeated at least three times.

### Statistical analysis

Statistical analyses were performed using ANOVA, or when the requirements were not met, by unpaired t‐test. Multiple comparison was performed using Holm-Sidak’s multiple comparisons test. *P*‐values of <0.05 were considered statistically significant. Normality and equal variance assumptions were tested with the Kolmogorov–Smirnov test and the Brown–Forsythe test, respectively. All analyses were performed using GraphPad Prism for Windows (version 9.02) software.

### Reporting summary

Further information on research design is available in the Nature Portfolio Reporting Summary linked to this article.

## Supplementary information

Supplementary Information

Peer Review File

Reporting Summary

## Data Availability

The data that support the findings of this study are available within the article and supplementary information. Source data are provided with this paper.

## Source data

Source Data
