# Supplementary material for: A trans-kingdom T6SS effector induces the fragmentation of the mitochondrial network and activates innate immune receptor NLRX1 to promote infection
Source: Nat Commun. 2023 Feb 16;14:871. doi: 10.1038/s41467-023-36629-3 (PMC9935632; doi:10.1038/s41467-023-36629-3)
Supplement: Supplementary file 1 — Supplementary Information [file 41467_2023_36629_MOESM1_ESM.pdf]

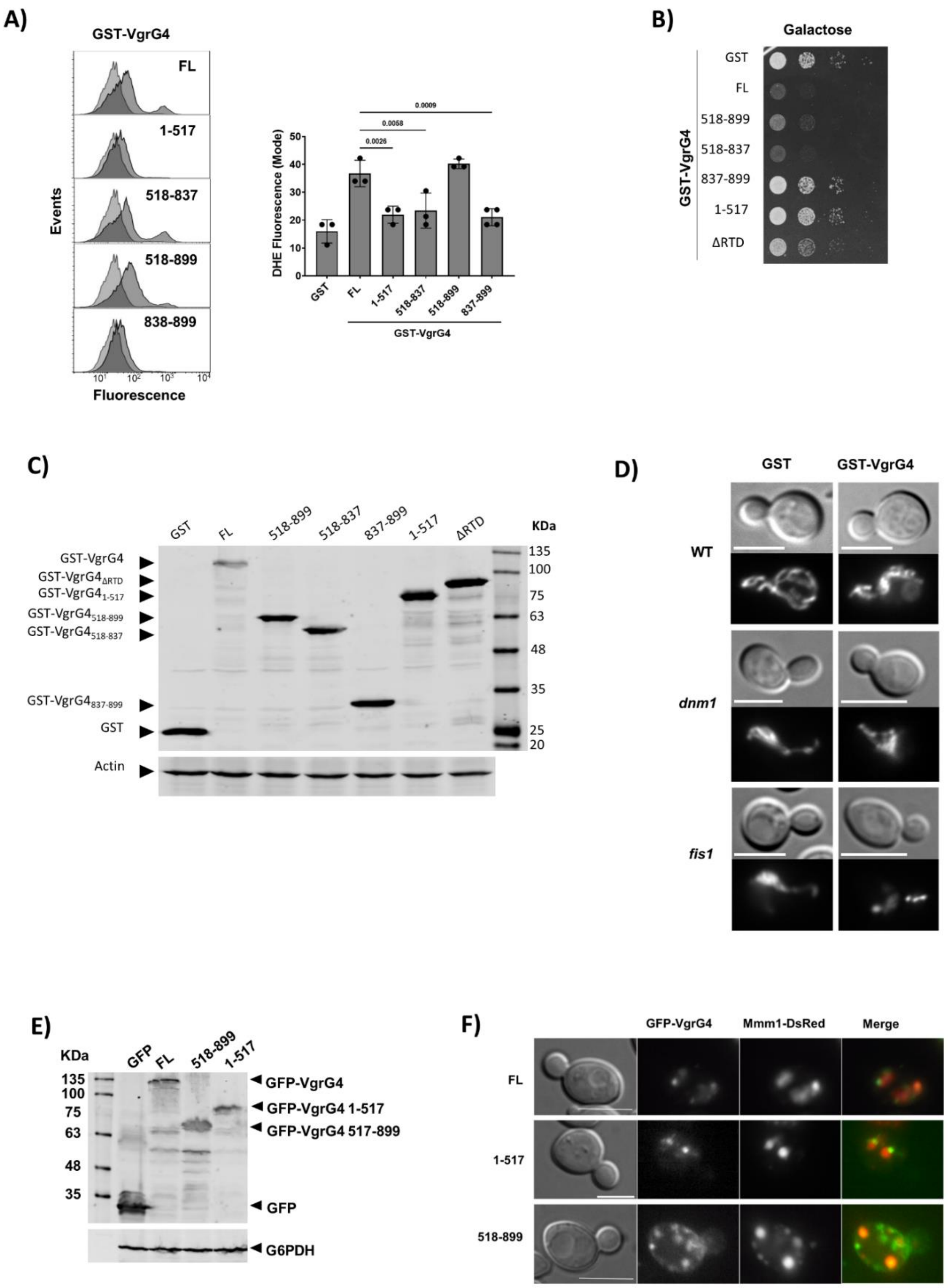

Supplementary Figure 1. VgrG4 triggers ROS production in yeast and causes mitochondrial condensation.

(A) Expression of VgrG4 RTD in yeast leads to ROS generation. Fusions to GST constructed in the pEG(KG) *GAL1*-driven expression vector of full-length (FL) *K. pneumoniae* VgrG4 or the indicated truncated versions were expressed in the YPH499 strain, and stained with dihydroethidium (DHE) to detect ROS production. Cultures were grown in expression conditions for 16 h and stained with DHE. The left panel shows histograms from a representative experiment. The white plot represents the control culture expressing GST alone, overlapped by the corresponding plots of the cultures expressing the indicated GST-VgrG4 fusions (in grey), as indicated. Shifting of the peak to the right with respect to control reflects ROS enhancement. The graph at

the right panel depicts the average mode of histograms from three independent experiments performed on different clones either GST alone (left panels) or GST-VgrG4 (right panels), with standard deviation error bars. Two-tailed student's T-test was applied for statistical significance (p values indicated in the graph), taking GST-VgrG4$_{1\text{-}517}$, lacking the RTD domain, as a reference.

(B) Toxicity of VgrG4 in yeast is partially alleviated by deletion of the RTD stretch. Drop growth assay of representative clones expressing GST as a control or the GST-VgrG4 fusions indicated. Decimal serial dilutions were spotted on galactose-based SG Ura$^-$ Leu$^-$ agar to induce expression from the *GAL1* promoter.

(C) Immunoblot showing the expression of the GST-VgrG4 fusions, full length (FL) or the indicated truncated versions. Lysates from yeast cells previously induced for 5 h in galactose-based media to express the heterologous protein, then subject to SDS-PAGE and immunoblotted with anti-GST rabbit polyclonal antibody (Z-5): sc-459 (Santa Cruz Biotechnology) diluted 1:5000. As a loading control, mouse anti-actin monoclonal antibodies (Clone C4, MP Biomedicals) diluted 1:1000 were used.

(D) Representative images of cells expressing the Ilv6-DsRed mitochondrial marker and either GST alone (left panels) of GST-VgrG4 (right panels). Wild type and mitochondrial fission mutants are shown expressing. VgrG4 expression leads to mitochondrial defects reminiscent of those displayed by *dnm1* or *fis1* deletants, and VgrG4 expression in these mutants does not alter their characteristic phenotype. Scale bars in microscopy images represent 5 µm.

(E) Immunoblot showing expression of the GFP VgrG4 fusions, either full length (FL) or truncated versions, as indicated. Lysates from yeast cells previously induced for 5 h in galactose to express the GFP fusions, were subject to SDS-PAGE and immunoblotted with anti-GFP antibody (Anti GFP-JL8, mouse monoclonal, Clontech) diluted 1:100. As a load control, rabbit anti-G6PDH antibodies (Sigma) were used at a 1:50,000 dilution.

(F) Partial co-localisation of VgrG4 and the truncated versions indicated (green channel) and Mmm1 (red channel). SLY001 cells were co-transformed with pAG424-GAL-Mmm1-DsRed and the pYES2-GFP-VgrG4 versions indicated, and observed by fluorescence microscopy. Scale bars represent 5 µm.

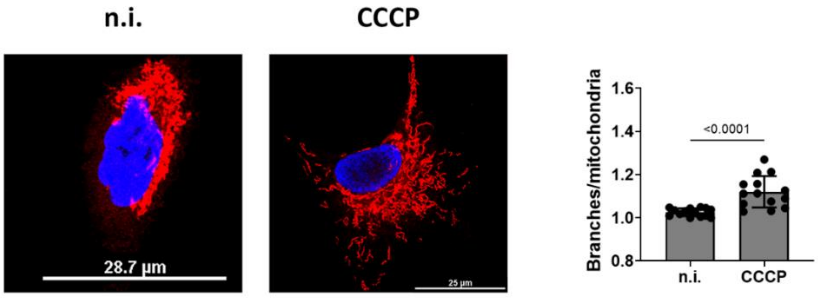

Supplementary Figure 2. CCCP causes mitochondria fragmentation.

Confocal microscopy of A549 cells treated with mitotracker red (50 µM, 30 min, in red) and stimulated with CCCP (10 µM, 30 min). Images are representative of three independent experiments. The number of branches/mitochondria was determined with the Mitochondria Analyzer plugin for ImageJ. The graph is the result of the analysis of minimum fifteen images per condition of three independent experiments representing average with standard deviation error bars; at least 100 cells were analysed per sample. One-way ANOVA with Tukey's multiple comparison test was applied for statistical significance (ns – non significant; **** p<0.0001).

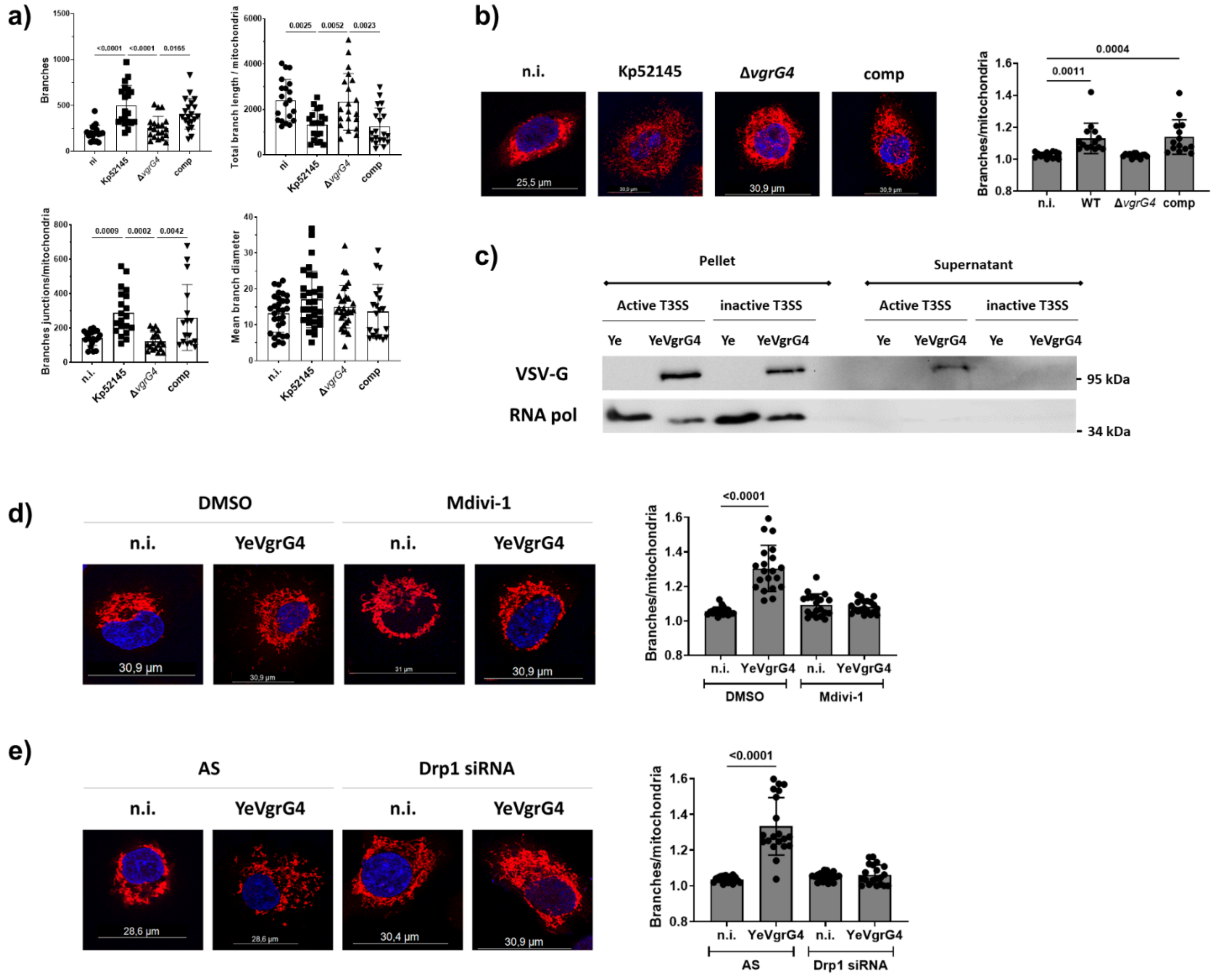

Supplementary Figure 3. Mitochondria fragmentation analysis.

(A) Branching of the mitochondria was determined with the Mitochondria Analyzer plugin for ImageJ. Graphs show the number of branches, total branch length/mitochondria, number of junctions/mitochondria and mean branch diameter. The graph is the result of the analysis of twenty images per condition from three independent experiments; at least 100 cells were analysed per sample. Data in graphs are presented as the mean ± SD. P values indicated in the graph using two way-ANOVA with Holm-Sidak's multiple comparisons test.

(B) Mitochondria fragmentation in NuLi-1 cells treated with mitotracker red (50 µM, 30 min, in red) and infected with either Kp52145, the *vgrG4* mutant (Δ*vgrG4*), or its complemented strain (comp, Δ*vgrG4*/pBAD30*vgrG4*) for 3 h. Nuclei were stained with Hoechst (DAPI, in blue). The number of branches/mitochondria was determined with the Mitochondria Analyzer plugin for ImageJ; at least 100 cells were analysed per sample. Data is presented representing average with standard deviation error bars. P values indicated in the graph; determined using one way-ANOVA with Holm-Sidak's multiple comparisons test.

(C) Immunoblot analysis of VSV-G and RNA polymerase levels in lysates and supernatants of *Y. enterocolitica* with active (calcium restriction), and inactive T3SS. Image is representative of three independent experiments.

(D) Confocal microscopy of A549 cells treated with the Drp1 inhibitor Mdivi-1 (10 µM, 2 h before infection) or DMSO (vehicle solution) and infected with YeVgrG4 for 90 min. Cells were pre-treated with mitotracker red (50 µM, 30 min, in red) and nuclei were stained with Hoechst (DAPI, in blue). Mitochondria fragmentation

was also analysed in cells transfected with a non-silencing control (AS – All Stars) or with a Drp1 siRNA, and treated with mitotracker red (50 µM, 30 min, in red), and infected with YeVgrG4 for 90 min (E). The number of branches/mitochondria was determined with the Mitochondria Analyzer plugin for ImageJ. The graph is the result of the analysis of twenty images per condition from three independent experiments; at least 100 cells were analysed per sample. Data is presented representing average with standard deviation error bars. P values indicated in the graph; determined using one way-ANOVA with Holm-Sidak's multiple comparisons test.

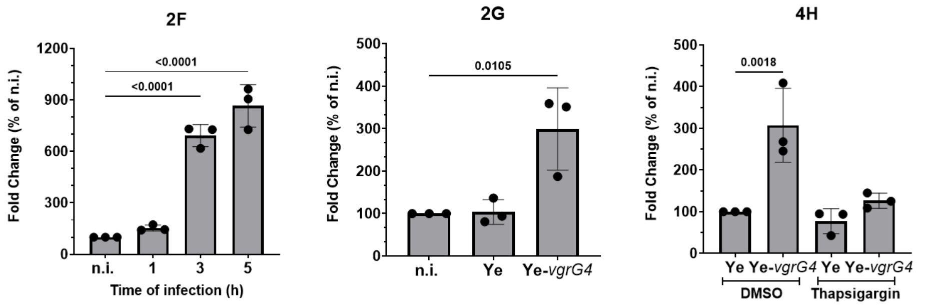

Supplementary Figure 4. Quantification of the Drp1 blots.

p-Drp1 blots corresponding to image Fig 2F (2F), Fig 2G (2G) and Fig 4H (4H) were quantified from three independent experiments using Image Studio Lite (Li-cor) and the modification normalised to total Drp1 signal. The graph represents average with standard deviation error bars of fold change (in %) compared to non-infected control cells. P values indicated in the graph; versus n.i. determined using one way-ANOVA with Tukey's multiple comparisons test.

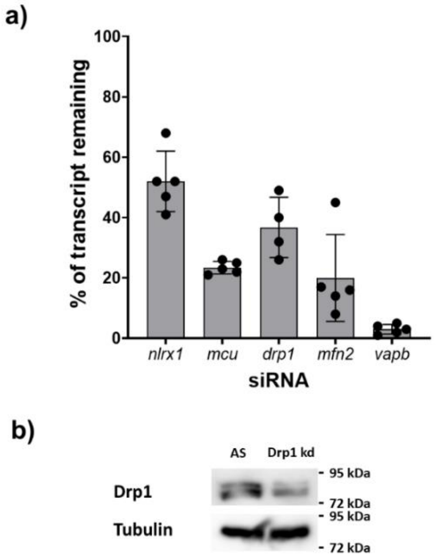

Supplementary Figure 5. Efficiency of transfection of siRNA into A549 cells.

(A) Efficiency of knockdown presented as percent (%) of transcript remaining post transfection. mRNA levels of the indicated transcripts were accessed 48 h post-transfection as fold change against control non-silencing agent (AS-AllStars control), after gene normalization. Values are presented as the mean ± SD of three independent experiments measured in duplicate.

(B) Immunoblot analysis of Drp1 and tubulin levels in lysates of A549 cells transfected with Drp1 siRNA (20 nM) or a non-silencing control (AS). Images are representative of three independent experiments.

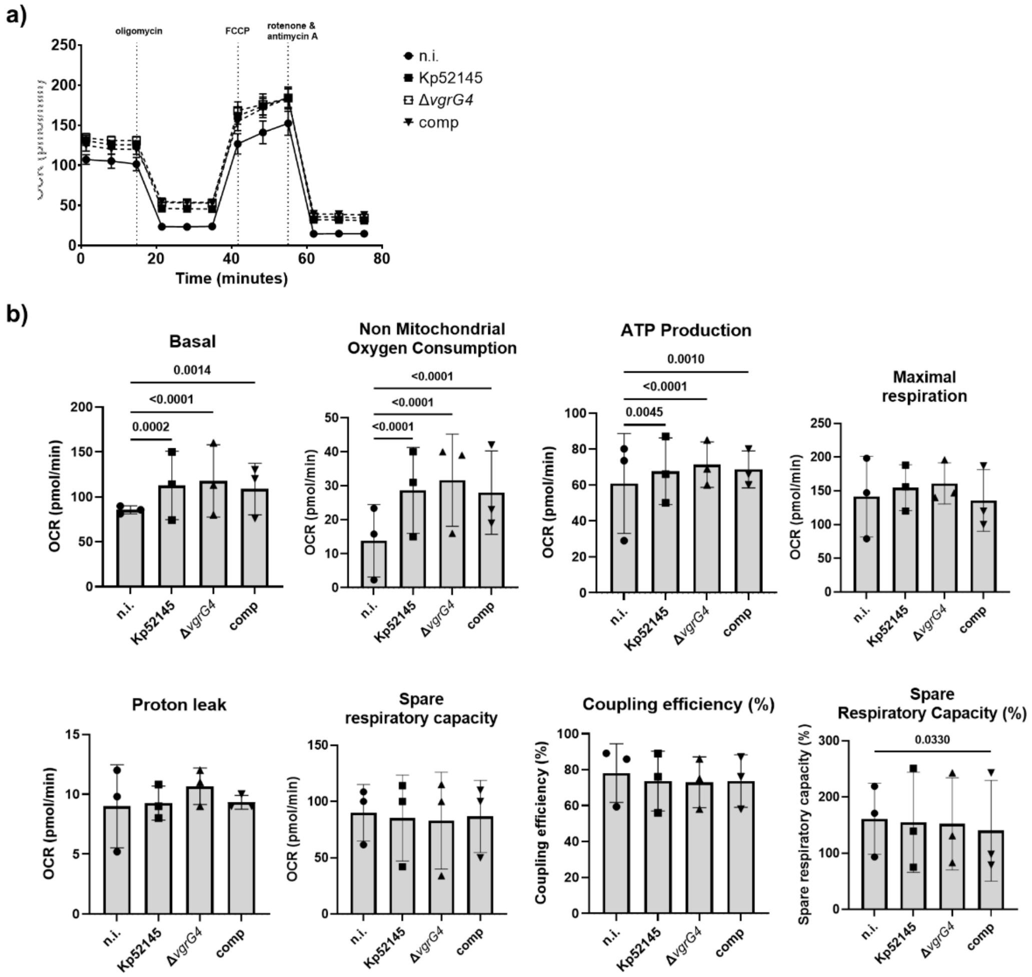

Supplementary Figure 6. Cellular bioenergetics of A549 infected cells.

(A) Oxygen consumption rates (OCR, in pMoles/min) by A549 cells measured using Mito-stress test kit and the Seahorse XF1580 analyser. Cells were infected with either Kp52145, the *vgrG4* mutant (strain Δ*vgrG4*), its complementation (comp, Δ*vgrG4*/pBAD30*vgrG4*) or left uninfected (n.i.) for 3 h. Values are presented as the mean ± SD of three independent experiments measured in triplicate. When indicated, oligomycin (2.5 µM), FCCP (2 µM), antimycin and roteanone (0.5 µM) were added to the cells.

(B) Basal respiration, non-mitochondrial oxygen consumption, ATP production, maximal respiration, proton leak, spare respiratory capacity, coupling efficiency and spare respiratory capacity of *K. pneumoniae* infected A549 cells obtained using the Mito-stress test kit in the Seahorse XF1580 analyser. Values are presented as the mean ± SD of three independent experiments. P values indicated in the graph were determined using one way-ANOVA with Holm-Sidak's multiple comparisons test.

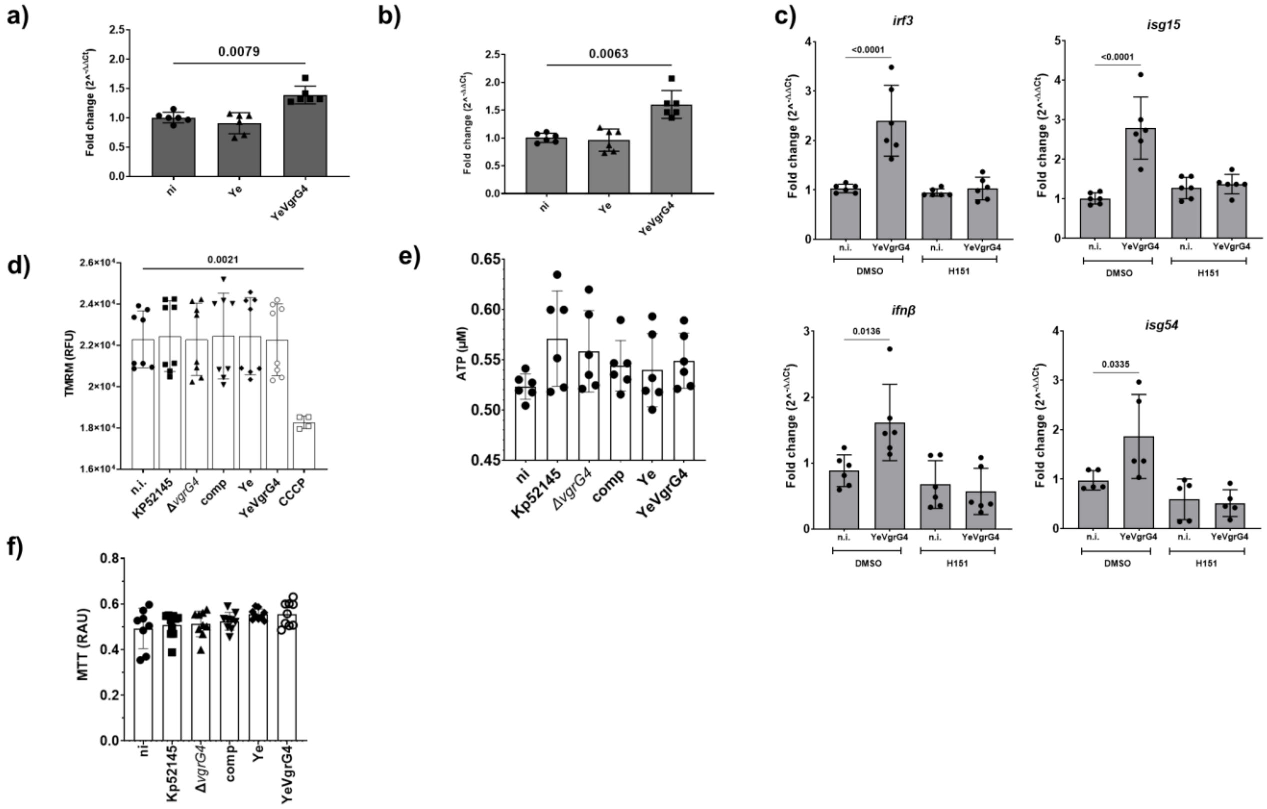

Supplementary Figure 7. VgrG4-induced mitochondrial fragmentation causes mtDNA release and STING activation.

(A) qPCR analysis of mitochondrial cytochrome c (mtCO-1) levels using polymerase β ribosomal DNA as housekeeping gene (ratio mitochondria/nuclear DNA) in the cytosol of A549 cells infected with *Y. enterocolitica* strains. Values are presented as the mean ± SD of three independent experiments measured in duplicate. P values indicated in the graph were calculated using one way-ANOVA with Tukey's multiple comparisons test.

(B) qPCR analysis of mitochondrial cytochrome c (mtCO-1) levels using polymerase β ribosomal DNA as housekeeping gene (ratio mitochondria/nuclear DNA) in the cytosol of NuLi-1 cells infected with *Y. enterocolitica* strains. Values are presented as the mean ± SD of three independent experiments measured in duplicate. P values indicated in the graph were calculated using one way-ANOVA with Tukey's multiple comparisons test.

(C) mRNA levels (fold change) of the interferon stimulated genes *irf3* (Interferon Regulatory Factor 3), *isg15* (Interferon-Stimulated Gene 15), *isg54* (interferon-stimulated gene 54) and *ifnβ* (Interferon β) assessed by RT-qPCR, in A549 cells left untreated (n.i.) or infected with YeVgrG4 for 90 min. Cells were pre-treated with the STING inhibitor H151 (5 µM, 2 h pre-infection) or a vehicle control (DMSO). Values are presented as the mean ± SD of three independent experiments measured in duplicate. P values indicated in the graph were calculated using one way-ANOVA with Tukey's multiple comparisons test.

(D) Mitochondria membrane potential was measured in A549 cells infected with *Y. enterocolitica* strains for 90 min, or *K. pneumoniae* strains for 3 h or left uninfected (n.i.). The uncoupler CCCP was used as a positive control (1 nM, 5 min). Membrane potential was determined using a TMRM kit and results expressed in relative fluorescence units. Values are presented as the mean ± SD of four independent experiments measured in duplicate. P values indicated in the graph were calculated using one way-ANOVA with Tukey's multiple comparisons test.

(E) ATP concentration (in µM) of A549 cells infected with *Y. enterocolitica* strains for 90 min or *K. pneumoniae* strains for 3 h or left uninfected (n.i.). Values are presented as the mean ± SD of three independent experiments measured in duplicate. P values indicated in the graph were calculated using one way-ANOVA with Tukey's multiple comparisons test.

(F) Cell viability was assayed using a 2,5-diphenyl-2H-tetrazolium bromide (MTT) assay in A549 cells infected with *Y. enterocolitica* strains for 90 min or *K. pneumoniae* strains for 3h or left uninfected (n.i.). Values are presented as the mean ± SD of three independent experiments measured in triplicate. P values indicated in the graph were calculated using one way-ANOVA with Tukey's multiple comparisons test.

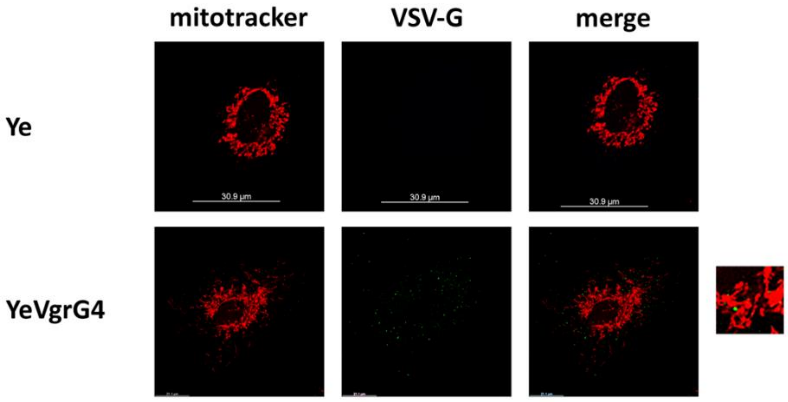

Supplementary Figure 8. VgrG4 does not colocalise with the mitochondria.

Confocal microscopy of A549 cells treated with mitotracker red (50 µM, 30 min, in red) and infected with *Y. enterocolitica* strains for 90 min. VSV-G staining was used to label VgrG4. Images are representative of three independent experiments.

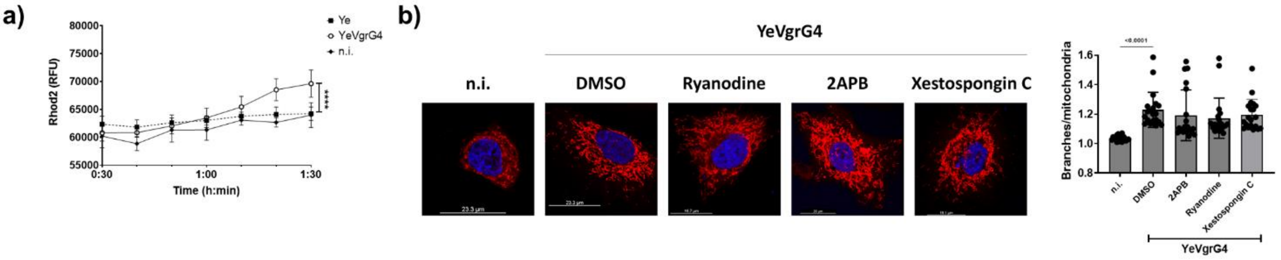

Supplementary Figure 9. VgrG4 triggers the transfer of Ca^{2+} from the ER to the mitochondria.

(A) Rhod2 fluorescence, in relative fluorescence units (RFU), was measured over the course of 1 hour in cells infected with *Y. enterocolitica* strains. Media was replaced by calcium free HBSS containing gentamicin (100 µg/mL) and Rhod2-AM (50 µM) added at 30 minutes post infection, and fluorescence was recorded every 20 min. Graph represents an average of 10 wells with standard deviation error bars.

(B) Confocal microscopy of A549 cells treated with mitotracker red (50 µM, 30 min, in red) and infected with YeVgrG4 for 90 min. Nuclei were stained with Hoechst (DAPI, in blue). Cells were treated with ryanodine (100 nM), 2APB (10 µM), Xestospogin C (10 µM) or vehicle control (DMSO, non treated) for 30 min. The number of branches/mitochondria was determined with the Mitochondria Analyzer plugin for ImageJ. The graph is the result of the analysis of twenty images per condition from three independent experiments; at least 100 cells were analysed per sample. Values are presented as the mean ± SD of three independent experiments measured in duplicate. P values indicated in the graph were calculated using one way-ANOVA with Tukey's multiple comparisons test.

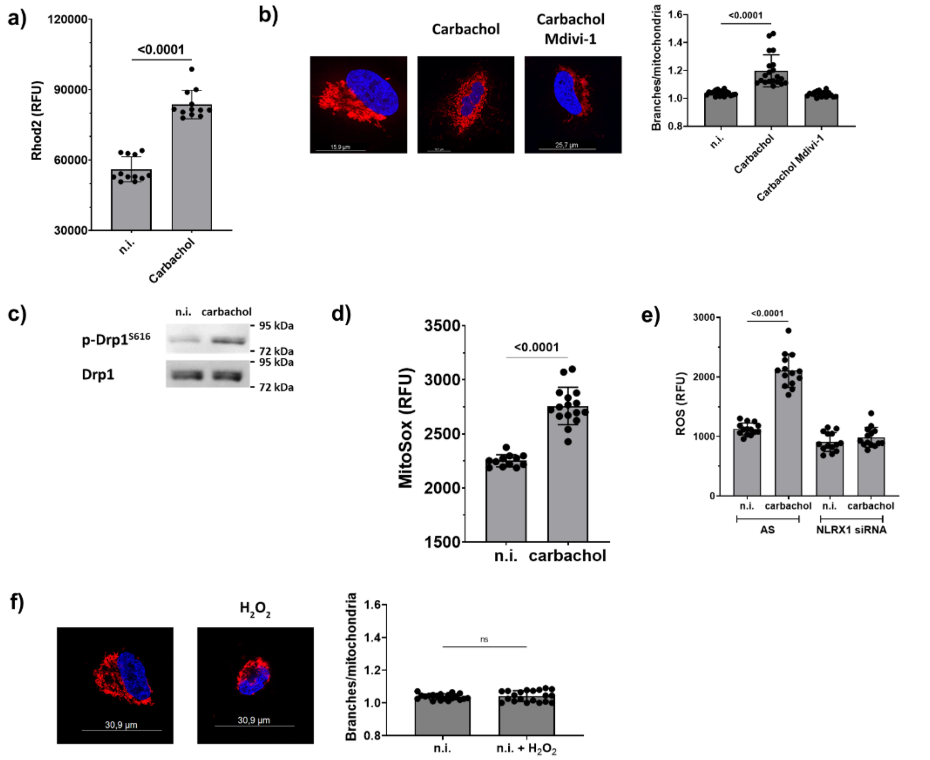

Supplementary Figure 10. Calcium induces mtROS but mtROS does not induce mitochondrial fragmentation.

(A) Rhod2-AM fluorescence was measured in non-infected cells treated with carbachol (10 µM, 30 min). (B) Mitochondria fragmentation was analysed in non-infected cells treated with either carbachol (10 µM, 30 min) alone or with a pre-treatment with Mdivi-1 (Drp1 inhibitor, 10 µM, 2 h). Cells were treated with mitotracker red (50 µM, 30 min, in red) and stained with Hoechst (DAPI, in blue) for nuclei identification. (C) Carbachol was enough to elicit phosphorylation of Drp1 as measured by immunoblot of non-stimulated cells or cells treated with carbachol (10 µM, 30 min) for phosphorylated Drp1 (S616) and using as loading control total Drp1 (D). Mitochondrial ROS, detected as fluorescence of the dye mitoSOX, was measured in A549 cells stimulated with carbachol (10 µM, 30 min in calcium free HBSS) and treated with mitoSOX red mitochondrial superoxide indicator (10 µM for 30 min). Values are presented as the mean ± SD of four independent experiments measured in triplicate. (E) DCF fluorescence was measured in A549 cells transfected with NLRX1 siRNA (50 nM) or a non-silencing control (AS) and treated with carbachol (10 µM, 30min). (F) Confocal microscopy of A549 cells treated with the ROS inducer hydrogen peroxide (H_2O_2, 5 µM, 15min). Mitochondria fragmentation was analysed in cells pre-treated with mitotracker red (50 µM, 30 min, in red) and stained with Hoechst (in blue).

Images and immunoblots are representative of three independent experiments. Data in graphs are presented as the mean ± SD of five independent experiments measured in duplicate. The number of branches/mitochondria was determined with the Mitochondria Analyzer plugin for ImageJ. The graph is the result of the analysis of minimum fifteen images per condition of three independent experiments representing average with standard deviation error bars; at least 100 cells were analysed per sample. t student and one-way ANOVA with Tukey's multiple comparison test was applied for statistical significance (P values indicated in the graph).

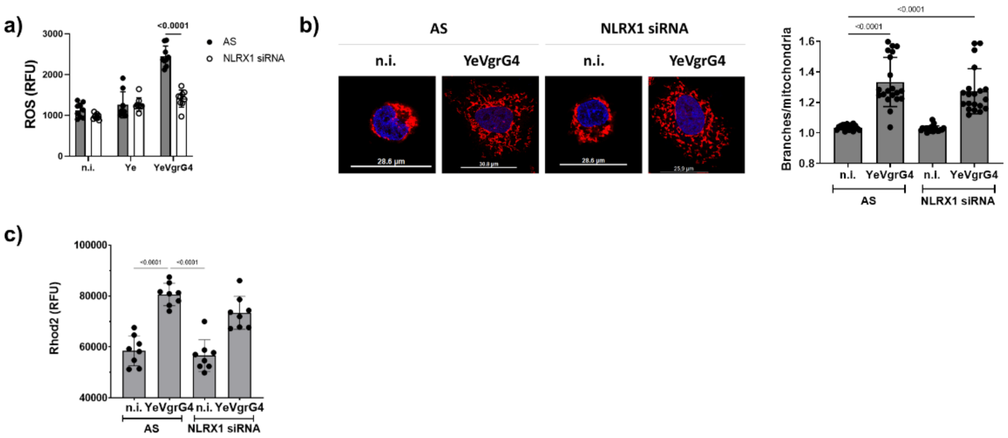

Supplementary Figure 11. VgrG4 is sufficient to induce mitochondrial ROS production upon activation of NLRX1.

(A) DCF fluorescence was measured in A549 cells transfected with NLRX1 siRNA (50 nM), and infected with *Y. enterocolitica* strains for 90 min. (B) Mitochondrial fragmentation was analysed in NLRX1 siRNA (50 nM) transfected cells infected with Ye-vgrG4 for 90 min. The number of branches/mitochondria was determined with the Mitochondria Analyzer plugin for ImageJ. The graph is the result of the analysis of twenty images per condition of three independent experiments representing average with standard deviation error bars; at least 100 cells were analysed per sample. (C) Rhod2-AM fluorescence was measured in NLRX1 siRNA transfected cells infected with YeVgrG4 for 90 min. One-way ANOVA with Tukey's multiple comparison test was applied for statistical significance (P values indicated in the graph). Images are representative of three independent experiments. Data in graphs are presented as the mean ± SD of three or four independent experiments measured in duplicate.

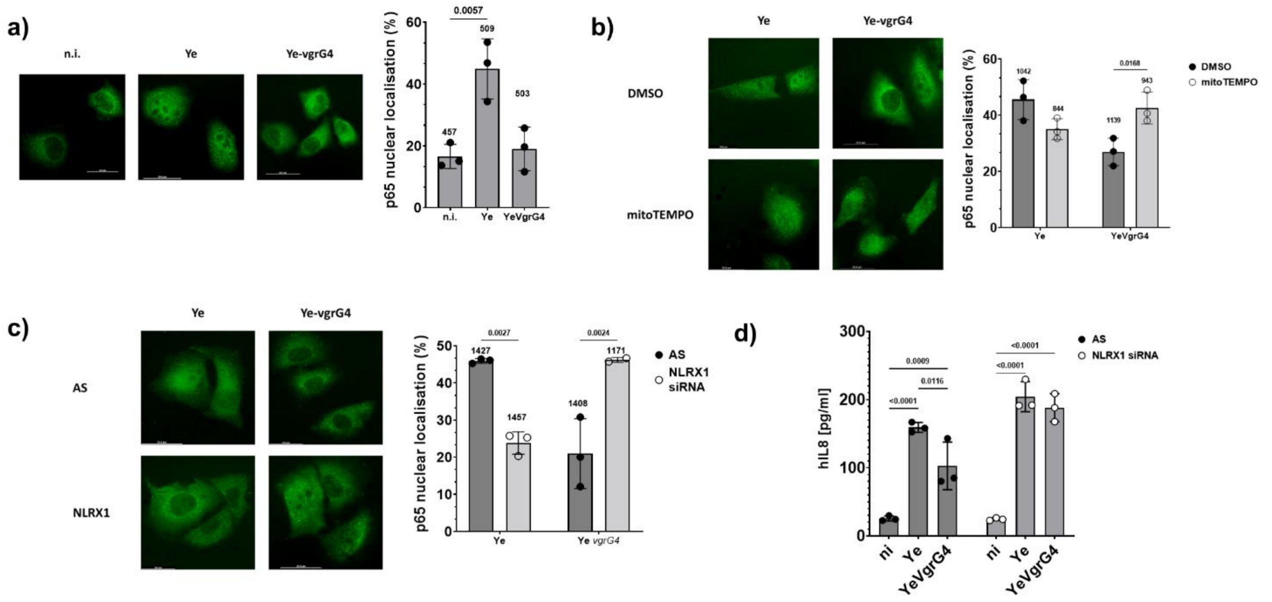

Supplementary Figure 12. NLRX1-mediated ROS induced by VgrG4 controls NF-κB signalling.

Immunofluorescence microscopy of A549 cells stained with antibody for the p65 NF-κB subunit. Cells were infected with *Y. enterocolitica* strains for 90 min (A), pre-treated with mitoTEMPO (10 µM, 2h pre-infection), or with vehicle control (DMSO) (B) or were transfected with NLRX1 siRNA (50 nM) or a non-silencing control (AS) (C). The % of p65 NF-κB localised in the nucleus is represented on the graph in A-E and is the result of counting of minimum of hundred cells from each of three independent experiments. The total number of counted cells is indicated on top of each bar. ELISA of IL-8 secreted by A549 cells transfected with either NLRX1 siRNA (50 nM) or a non-silencing control (AS), and infected with *Y. enterocolitica* strains for 30 min, after which the medium was replaced with medium containing gentamicin (100 µg/mL), and after 24 h the medium was collected (D). Images are representative of three independent experiments. Data in graphs are presented as the mean ± SD of three independent experiments. Two way-ANOVA with Holm-Sidak's multiple comparisons test was used for statistical significance (P values indicated in the graph).

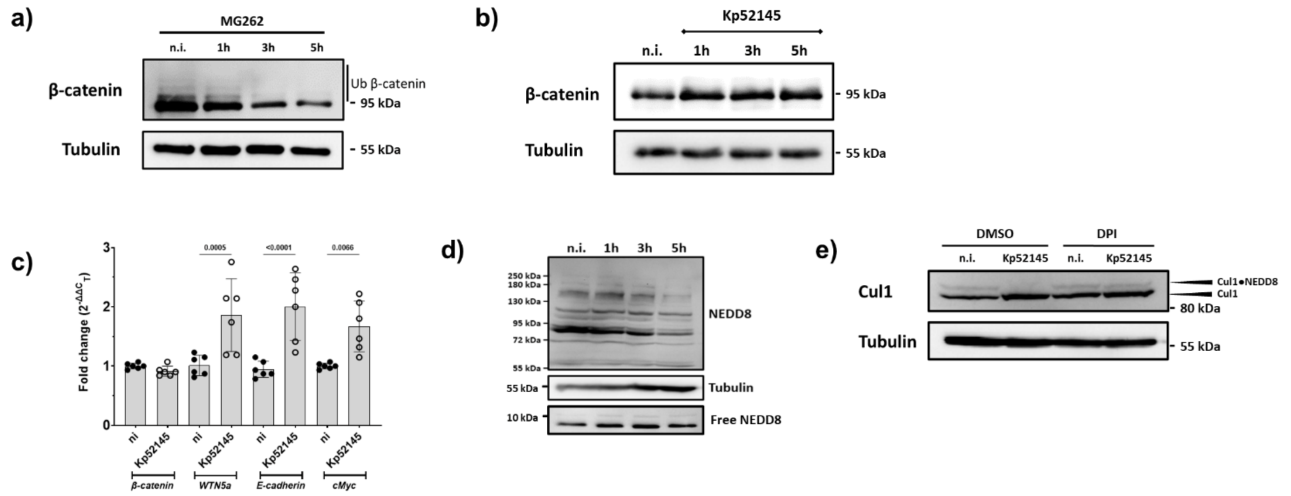

Supplementary Figure 13. ***K. pneumoniae*** **infection impairs β-catenin degradation.**

(A) Immunoblot analysis of β-catenin and tubulin levels in lysates of A549 cells infected with Kp52145 for the indicated time. Cells were pre-treated with the proteasome inhibitor MG262 (5 µM, 2 h before infection). The ubiquitin chains linked to the protein are labelled. Images are representative of three independent experiments.

(B) Immunoblot analysis of β-catenin and tubulin levels in lysates of A549 cells infected with Kp52145 for the indicated time. Images are representative of three independent experiments.

(C) mRNA levels (fold change) of *β-catenin* and downstream genes *WTN5a* (Wingless-Type MMTV Integration Site Family Member 5A), *E-cadherin*, *cMyc* (cellular Myelocytomatosis) assessed by qPCR, in A549 cells left untreated (n.i.) or infected with Kp52145 for 3 h. Values are presented as the mean ± SD of three independent experiments measured in duplicate. P values indicated in the graph were calculated using one way-ANOVA with Tukey's multiple comparisons test.

(D) Immunoblot analysis of NEDDylated proteins (NEDD8), the free NEDD8 protein (free NEDD8) and tubulin levels in lysates of A549 cells infected with Kp52145 for the indicated times. Images are representative of three independent experiments.

(E) Immunoblot analysis of Cul-1 and tubulin levels in lysates of A549 cells treated with vehicle control DMSO or DPI (500 µM, 1 h before infection), and infected with Kp52145 for 5 h. Images are representative of three independent experiments.

Supplementary Table 1. Strains and plasmids used in this study.

Strain or plasmid	Genotype or comments	Source or reference(s)
	Saccharomyces cerevisiae	
YPH499	*MATa ade 2-101 trp1-63 leu2-1 ura3-52 his3-200 lys2-801*	1
SLY001	*MAT*a/α *his3Δ1/his3Δ1 leu2Δ0/leu2Δ0 met15Δ0/met15Δ0 trp1Δ/trp1Δ ura3Δ0/ura3Δ0*	
VHY87	*MAT*α *leu 2-3 ura3-52 his4 can1^{R} TRP1*::DsRed-HDEL	
BY4741 *dnm1Δ*	*MAT*α *his3Δ1 leu2Δ0 met15Δ0 ura3Δ0 dnm1Δ::kanMX4*	Euroscarf
BY4741 *fus1Δ*	*MAT*α *his3Δ1 leu2Δ0 met15Δ0 ura3Δ0 fus1Δ::kanMX4*	Euroscarf
	Escherichia coli	
DH5α	F′ K12 Δ(l*ac*ZYA-*arg*F) U169 *deo*R *supE44 thi-1 rec*A1 *end*A1 *hsd*R17 *gyr*A96 *rel*A1 (φ80*lac*ZΔM15)	Laboratory stock
β2163	(F^{-}) RP4-2-Tc::Mu Δ*dapA*::(*erm-pir*) [Km^{R}Em^{R}]	2
	Klebsiella pneumoniae	
CIP52.145	Clinical isolate; serotype O1:K2; sequence type ST66	3
MGH78578	(ATCC700721) Clinical isolate; serotype O1:K2; sequence type ST38	ATCC
NTUH-K2044	Clinical isolate; serotype O1:K2; sequence type ST23	4
ATCC43816	Clinical isolate; serotype O1:K2; sequence type ST493	ATCC
SGH10	(NCTC 14052) Clinical isolate; serotype O1/O2.v2:K1; sequence type ST23	NCTC 14052
52145-Δ*vgrG4*	Kp52145, Δ*vgrG4*; T6SS *vgrG4* gene was inactivated	5
52145-Δ*clpV*	Kp52145, Δ*clpV*; *clpV* gene of T6SS cluster I was inactivated	5
52145-comp	Kp52145, Δ*vgrG4*, expressing a pBAD30 plasmid containing the gene coding for *vgrG4*; Amp^{R}	5
vgrG4-FLAG	Kp52145, Δ*vgrG4*, expressing pBAD*vgrG4*-FLAG; Amp^{R}	This work
52145-*vgrG4*-GSK3β	Kp52145, expressing pBAD*vgrG4*-GSK3β; Amp^{R}	This work
ΔclpV-*vgrG4*-GSK3β	Kp52145, Δ*clpV*, expressing pBAD*vgrG4*-GSK3β; Amp^{R}	This work
	Yersinia enterocolitica	
Ye	Plasmidless derivative of strain WA-314 serotype O:8, harbouring the pT3SS plasmids; Spec^{R}	6
Ye *vgrG4*	Ye strain harbouring the plasmids pT3SS and pE$_{53}$-*vgrG4* with a VSVG tagged *vgrG4*; Spec^{R} Cm^{R}	This work
Ye *vgrG4* (1-517)	Ye strain harbouring the plasmids pT3SS and pE$_{53}$-*vgrG4*(1-517) with a VSVG tagged *vgrG4* truncated at amino acid 517; Spec^{R} Cm^{R}	This work
Ye *vgrG4* (518-C)	Ye strain harbouring the plasmids pT3SS and pE$_{53}$-*vgrG4*(518-C) with a VSVG tagged C-terminal *vgrG4* (from amino acid 518); Spec^{R} Cm^{R}	This work
	Plasmids	
pYES2-GFP	*URA3*-based yeast expression episomal plasmid for N-terminal GFP fusions, under *GAL1* promoter.	7
pYES2-GFP-VgrG4 FL	N-terminal GFP fusion to *K. pneumoniae* VgrG4 full length (FL)- for yeast expression under *GAL1* promoter	This work

pYES2-GFP-VgrG4 518-C	N-terminal GFP fusion to VgrG4 (518-899) fragment for yeast expression under *GAL1* promoter	This work
pAG424-GAL-Mdm34-DsRed	*S. cerevisiae* Mdm34 ERMES protein- DsRed fusion for yeast expression under *GAL1* promoter	Julia Maria Coronas Serna
pAG424-GAL-Mmm1-DsRed	*S. cerevisiae* Mmm1 ERMES protein- DsRed fusion for yeast expression under *GAL1* promoter	Julia Maria Coronas Serna
pAG413-GPD-Mdm34-HA	Mdm34 ERMES protein-HA fusion for yeast expression under GPD promoter.	This work
pAG413-GPD-Mmm1-HA	Mmm1 ERMES protein-HA fusion for yeast expression under GPD promoter.	This work
pYE-lac112-Ilv6-Cherry	*TRP1*-based plasmid expressing the Ilv6-mCherry mitochondrial protein, under the *ILV6* promoter	8
pEG(KG)-GST	*URA3-leu2d*-based episomal plasmid for N-terminal GST fusions, for yeast expression under *GAL1* promoter.	9
pEG(KG)-GST-VgrG4 FL	N-terminal GST fusion to *K. pneumoniae* VgrG4 full length (FL)-, for yeast expression under *GAL1* promoter.	5
pEG(KG)-GST-VgrG4 518-899	N-terminal GST fusion of VgrG4 (518-899) fragment for yeast expression under *GAL1* promoter	5
pEG(KG)-GST-VgrG4ΔRTD	N-terminal GST fusion of VgrG4 without the RTD fragment for yeast expression under *GAL1* promoter	This work
YEp-lac112-Ilv6-Cherry	Organellar marker to visualize the mitochondria	10
pBAD30	pACYC184/p15A Origin of Replication, expression construct; Amp^R	11
pBAD*vgrG4*	pBAD30 plasmid containing the gene coding for the Kp52145 T6SS protein *vgrG4*; Amp^R	5
pBAD*vgrG4*-FLAG	pBAD30 plasmid containing the Kp52145 *vgrG4* fused with a FLAG tag; Amp^R	5
pBAD*vgrG4*-GSK3β	pBAD30 plasmid containing the Kp52145 *vgrG4* fused with a GSK3β tag; Amp^R	This work
pE_{53}	*Hind*III–*Sal*I *sycE-yopE*$_{53}$– *ipgB*$_1$ fragment in pACYC184 (containing 53 codons of the *yopE* gene)	6
pE_{53}-*vgrG4*	*Bam*HI–*Sal*I *ipgB*$_1$ fragment in pE_{53} was replaced with Kp52145 *Bam*HI-*Sal*I *vgrG4* fragment and fused with a VSV-G tag	This work
pE_{53}-*vgrG4*(1-517)	*Bam*HI–*Sal*I *ipgB*$_1$ fragment in pE_{53} was replaced with Kp52145 *Bam*HI-*Sal*I *vgrG4* fragment truncated at the codon for amino acid 517 and fused with a VSV-G tag	This work
pE_{53}-*vgrG4*(518-C)	*Bam*HI–*Sal*I *ipgB*$_1$ fragment in pE_{53} was replaced with Kp52145 *Bam*HI-*Sal*I *vgrG4* fragment from the codons for amino acid 518 until 899 and fused with a VSV-G tag	This work

Supplementary Table 2. Primers used in this study.

Name	Sequence (5'-3')
UP-VgrG4-FL	GCA GAT CTA TGG ATA CCT CTT CAA TA
UP-VgrG4-518	CGA GAT CTG GCA AGA CGC AGC TGA AC
UP-1-517XbaI	GCTCTAGAGATGGATACCTCTTCAATAATTAC
LO-1-517overlap838	CGGGTATCTGATAGCTGACGCCGTAATCGGTGCTGAGC
UP-838overlap517	GCTCAGCACCGATTACGGCGTCAGCTATCAGATACCCG
LO-838-899XbaI	GCTCTAGATCATTGACTTTCGTCCTGTTC
LO-VgrG4	GCA GAT CTT CAT TGA CTT TCG TCC TG
(1-517)-1 (mutation in bold)	TCA GCA CCG ATT ACG GC**T GA**A AGA CGC AGC TGA ACC
(1-517)-2 (mutation in bold)	GGT TCA GCT GCG TCT T**TC A**GC CGT AAT CGG TGC TGA
GSK3b_F1	TTT CTG CAG ATG AGT GGT CGC CCT CGC ACT ACT AGT TTC GCT GAA AGT TGA CTC GAG TTT
GSK3b_R1	AAA CTC GAG TCA ACT TTC AGC GAA ACT AGT AGT GCG AGG GCG ACC ACT CAT CTG CAG AAA
Kpn52_*vgrG4*_VSVG_F1	GCG AGA TCT ATG GAT ACC TCT TCA ATA ATT A
Kpn52_*vgrG4*_VSVG_R1	CGC CTC GAG TCA CTT ACC CAG GCG GTT CAT TTC GAT ATC AGT GTA TTG ACT TTC GTC CTG TTC AA
Kpn52_*vgrG4*_VSVG_R2	CGC CTC GAG TCA CTT ACC CAG GCG GTT CAT TTC GAT ATC AGT GTA GCC GTA ATC GGT GCT GAC
Kpn52_*vgrG4*_518-C	GCG AGA TCT GGC AAG ACG CAG CTG AAC
h*MCU* forward	GGT AGA TCG CTC CTG CTG CTC
h*MCU* reverse	AGG AAG CGA TCC TCT GGT GTA
h*NLRX1* forward	GTG CCC GGA AGC TGG GCT TG
h*NLRX1* reverse	CCG GGC ACC ACC TTC AGC AG
h*VAPB* forward	AGC GGA ATC ATC GAT GCA GG
h*VAPB* reverse	TTT GCC TCC TTC CAT ACT GCT
h*MFN2* forward	TCT CCC GGC CAA ACA TCT TC
h*MFN2* reverse	ACC AGG AAG CTG GTA CAA CG
h*DRP1* forward	GCT CCA GGA CGT CTT CAA CA
h*DRP1* reverse	TCT GCT TCC ACC CCA TTT TCT
h*GAPDH* forward	GAG AAG GCT GGG GCT CAT TT
h*GAPDH* reverse	AGT GAT GGC ATG GAC TGT GG
h*IRF3* forward	AAG AAG GGT TGC GTT TAG CA
h*IRF3* reverse	TCC CCA ACT CCT GAG TTC AC
h*ISG15* forward	TTT GCC AGT ACA GGA GCT TGT G
h*ISG15* reverse	GGG TGA TCT GCG CCT TCA
h*IFNβ* forward	CTT TGC TCT GGC ACA ACA GG
h*IFNβ* reverse	GTG GAG AAG CAC AAC AGG AGA
h*ISG54* forward	GCA CTG CAA CCA TGA GTG AGA A
h*ISG54* reverse	TTC TCC CTC CAT CAA GTT CCA G
h*mtCO-1* forward	ATA TTT CAC CTC CGC TAC CA
h*mtCO-1* reverse	TCA GCT AAA TAC TTT GAC GCC
h*POLβ* forward	CGT GAA GAG ATG TTA CAA ATG C
h*POLβ* reverse	CAG ACT GTA GCA ATG TAT TCA G
hβCATENIN (*CTNNB1*) forward	AAA ATG GCA GTG CGT TTA G
hβCATENIN (*CTNNB1*) reverse	TTT GAA GGC AGT CTG TCG TA
h*WTN5a* forward	CGG TGT ACA ACC TGG CTG ATG
h*WTN5a* reverse	CACCTTGCGGAAGTCTGCC
h*E-CADHERIN* forward	CGG GAA TGC AGT TGA GGA TC
h*E-CADHERIN* reverse	AGG ATG GTG TAA GCG ATG GC
h*C-MYC* forward	CGT CCT CGG ATT CTC TGC TC
h*C-MYC* reverse	GCT GCG TAG TTG TGC TGA TG

Supplementary References

1 Sikorski, R. S. & Hieter, P. A system of shuttle vectors and yeast host strains designed for efficient manipulation of DNA in *Saccharomyces cerevisiae*. *Genetics* 122, 19-27, (1989).

2 Demarre, G. *et al.* A new family of mobilizable suicide plasmids based on broad host range R388 plasmid (IncW) and RP4 plasmid (IncPalpha) conjugative machineries and their cognate *Escherichia coli* host strains. *Res Microbiol* 156, 245-255, (2005).

3 Nassif, X. & Sansonetti, P. J. Correlation of the virulence of *Klebsiella pneumoniae* K1 and K2 with the presence of a plasmid encoding aerobactin. *Infection and immunity* 54, 603-608 (1986).

4 Wu, K. M. *et al.* Genome sequencing and comparative analysis of *Klebsiella pneumoniae* NTUH-K2044, a strain causing liver abscess and meningitis. *Journal of Bacteriology* 191, 4492-4501, (2009).

5 Storey, D. *et al. Klebsiella pneumoniae* type VI secretion system-mediated microbial competition is PhoPQ controlled and reactive oxygen species dependent. *PLoS Pathog* 16, e1007969, (2020).

6 Wolke, S., Ackermann, N. & Heesemann, J. The *Yersinia enterocolitica* type 3 secretion system (T3SS) as toolbox for studying the cell biological effects of bacterial Rho GTPase modulating T3SS effector proteins. *Cellular microbiology* 13, 1339-1357, (2011).

7 Rodriguez-Escudero, I., Andres-Pons, A., Pulido, R., Molina, M. & Cid, V. J. Phosphatidylinositol 3-kinase-dependent activation of mammalian protein kinase B/Akt in *Saccharomyces cerevisiae*, an in vivo model for the functional study of Akt mutations. *J Biol Chem* 284, 13373-13383, (2009).

8 Fernandez-Acero, T. *et al.* Expression of Human PTEN-L in a Yeast Heterologous Model Unveils Specific N-Terminal Motifs Controlling PTEN-L Subcellular Localization and Function. *Cells* 8, (2019).

9 Mitchell, D. A., Marshall, T. K. & Deschenes, R. J. Vectors for the inducible overexpression of glutathione S-transferase fusion proteins in yeast. *Yeast* 9, 715-722, (1993).

10 Huh, W. K. *et al.* Global analysis of protein localization in budding yeast. *Nature* 425, 686-691, (2003).

11 Guzman, L. M., Belin, D., Carson, M. J. & Beckwith, J. Tight regulation, modulation, and high-level expression by vectors containing the arabinose PBAD promoter. *Journal of Bacteriology* 177, 4121-4130 (1995).
