# Supplementary material for: A trans-kingdom T6SS effector induces the fragmentation of the mitochondrial network and activates innate immune receptor NLRX1 to promote infection
Source: Nat Commun. 2023 Feb 16;14:871. doi: 10.1038/s41467-023-36629-3 (PMC9935632; doi:10.1038/s41467-023-36629-3)
Supplement: Supplementary file 3 — Reporting Summary [file 41467_2023_36629_MOESM3_ESM.pdf]

nature portfolio

Corresponding author(s): Prof Jose Bengoechea

Last updated by author(s): 03/02/2023

Reporting Summary

Nature Portfolio wishes to improve the reproducibility of the work that we publish. This form provides structure for consistency and transparency in reporting. For further information on Nature Portfolio policies, see our Editorial Policies and the Editorial Policy Checklist.

Statistics

For all statistical analyses, confirm that the following items are present in the figure legend, table legend, main text, or Methods section.

n/a	Confirmed	
☐	☒	The exact sample size (*n*) for each experimental group/condition, given as a discrete number and unit of measurement
☐	☒	A statement on whether measurements were taken from distinct samples or whether the same sample was measured repeatedly
☐	☒	The statistical test(s) used AND whether they are one- or two-sided<br>*Only common tests should be described solely by name; describe more complex techniques in the Methods section.*
☐	☒	A description of all covariates tested
☐	☒	A description of any assumptions or corrections, such as tests of normality and adjustment for multiple comparisons
☐	☒	A full description of the statistical parameters including central tendency (e.g. means) or other basic estimates (e.g. regression coefficient) AND variation (e.g. standard deviation) or associated estimates of uncertainty (e.g. confidence intervals)
☐	☒	For null hypothesis testing, the test statistic (e.g. *F*, *t*, *r*) with confidence intervals, effect sizes, degrees of freedom and *P* value noted<br>*Give P values as exact values whenever suitable.*
☒	☐	For Bayesian analysis, information on the choice of priors and Markov chain Monte Carlo settings
☒	☐	For hierarchical and complex designs, identification of the appropriate level for tests and full reporting of outcomes
☒	☐	Estimates of effect sizes (e.g. Cohen's *d*, Pearson's *r*), indicating how they were calculated

Our web collection on statistics for biologists contains articles on many of the points above.

Software and code

Policy information about availability of computer code

Data collection	Images were collected with Leica SP8 laser scanning confocal microscope, and an Eclipse TE2000U microscope (Nikon, Tokyo, Japan) and digital images were acquired with an Orca C4742-95-12ER charge-coupled-device camera (Hamamatsu Photonics, Hamamatsu City, Japan).<br>Chemiluminescence was recorded with G:BOX Chemi XRQ chemiluminescence imager (Syngene).<br>Fluorescence was captured using Odyssey Infrared Imaging System (Li-Cor Biosciences).<br>Yeast cells were analysed using a 419 FACScan (Becton Dickinson) flow cytometer through a 488 nm excitation laser and a 585/42 BP emission filter (FL2).
Data analysis	Acquired images were analysed using the LAS imaging software (version 3.7, Leica), and HC Image (Hamamatsu).<br>Mitochondrial morphology was performed using the plugin for ImageJ Mitochondria Analyzer in 20 images for each condition from at least three independent experiments (https://github.com/AhsenChaudhry/Mitochondria-Analyzer).<br>Densitometry analysis of blots was done using Image Studio Lite version 5.2 (Li-cor).<br>Flow data was processed using FlowJo software version V10CL (FlowJo LLC, Ashland, OR, USA)<br>Statistical analysis was performed using GraphPad Prism for Windows (version 9.02) software.

For manuscripts utilizing custom algorithms or software that are central to the research but not yet described in published literature, software must be made available to editors and reviewers. We strongly encourage code deposition in a community repository (e.g. GitHub). See the Nature Portfolio guidelines for submitting code & software for further information.

Data

Policy information about availability of data

All manuscripts must include a data availability statement. This statement should provide the following information, where applicable:

- Accession codes, unique identifiers, or web links for publicly available datasets
- A description of any restrictions on data availability
- For clinical datasets or third party data, please ensure that the statement adheres to our policy

All data supporting the findings of this study are provided within the figures, supplementary material file, and source data file.

Human research participants

Policy information about studies involving human research participants and Sex and Gender in Research.

Reporting on sex and gender	N/A
Population characteristics	N/A
Recruitment	N/A
Ethics oversight	N/A

Note that full information on the approval of the study protocol must also be provided in the manuscript.

Field-specific reporting

Please select the one below that is the best fit for your research. If you are not sure, read the appropriate sections before making your selection.

- [x] Life sciences
- [ ] Behavioural & social sciences
- [ ] Ecological, evolutionary & environmental sciences

For a reference copy of the document with all sections, see nature.com/documents/nr-reporting-summary-flat.pdf

Life sciences study design

All studies must disclose on these points even when the disclosure is negative.

Sample size	No statistical methods were used to predetermine sample size but a minimum of three independent experiments were performed and all samples sizes are indicated in figure legends.
Data exclusions	No data were excluded.
Replication	Experiments were replicated independently three times and the findings were reproducible.
Randomization	Cells were randomized for interventions.
Blinding	Researches processing the samples and analysing the data were aware which intervention group corresponded to which sample group. Samples were quantified using an automatic plugin removing the bias from absence of blinding.

Reporting for specific materials, systems and methods

We require information from authors about some types of materials, experimental systems and methods used in many studies. Here, indicate whether each material, system or method listed is relevant to your study. If you are not sure if a list item applies to your research, read the appropriate section before selecting a response.

Materials & experimental systems

n/a	Involved in the study	
☐	☒	Antibodies
☐	☒	Eukaryotic cell lines
☒	☐	Palaeontology and archaeology
☒	☐	Animals and other organisms
☒	☐	Clinical data
☒	☐	Dual use research of concern

Methods

n/a	Involved in the study	
☒	☐	ChIP-seq
☐	☒	Flow cytometry
☒	☐	MRI-based neuroimaging

Antibodies

Antibodies used

anti-VSVG (1:1000, Sigma V4888)
anti-phospho-GSK3β (1:1000, Cell Signaling Technology (CST) 5558)
anti-GSK3β tag (1:1000, CST 9325)
anti-GSK3β (1:1000, Santa Cruz Biotechnology sc-67075)
anti-phospho-DRP1 (Ser616) (1:1000, CST 4494)
anti-Drp1 (1:1000 CST 8570)
anti-Cullin-1 (1:200, Santa Cruz Biotechnology sc-12761)
anti-NEDD8 (1:1000, Invitrogen 34-1400)
anti-Ubc12 (1:1000, Santa Cruz Biotechnology sc-366017)
anti-β-catenin (1:1000, Santa Cruz Biotechnology sc-7199)
anti-K48 Polyubiquitin (1:1000, CST 4289)
anti-IκBα (1:1000, CST 4814)
anti-phospho-Iκκαβ (1:1000, CST 2697)
anti-phospho-IκBα (1:1000, Santa Cruz Biotechnology sc-7977)
anti-GFP (1:5000, Proteintech 66002)
anti-E.coli RNA polymerase α (1:5000, BioLegend 663102)
horseradish peroxidase-conjugated goat anti-rabbit immunoglobulins (1:5000, BioRad 170-6515)
goat anti-mouse immunoglobulins (1:5000, BioRad 170-6516)
mouse anti-goat immunoglobulins (1:5000, Santa Cruz Biotechnology sc-2354)
anti-human tubulin (1:3000, Sigma T5168)
Anti-HA antibody (1:1000, Anti-HA High Affinity, Rat monoclonal antibody (clone 3F10), Sigma-Aldrich)
anti-GST antibody (1:5000, Anti-GST rabbit polyclonal Antibody (Z-5): sc-459 (Santa Cruz Biotechnology)
IRDye800 anti-rat (Li-Cor Biosciences)
Alexa-680 anti-mouse (Invitrogen)
anti-Tom20 (1:500, Santa Cruz Biotechnology F-10 sc-17764)
anti-calnexin (1:1000, Santa Cruz Biotechnology AF-18 sc-23954)
anti-NF-κB p65 (1:400, Santa Cruz Biotechnology sc-372)
Alexa Fluor 488 (1:400, Life-tech A21206)
Alexa Fluor 647 (1:400, Life-tech A21244)
Anti-flag M2 (1:5000, Sigma F3165)
Alexa Fluor 488 (1:400, Abcam ab150117)
anti-GST rabbit polyclonal antibody (Z-5) (1:5000, Santa Cruz Biotechnology sc-459)
anti-actin (1:1000, Clone C4, MP Biomedicals 08691001)
anti-GFP antibody (1:100, Anti GFP-JL8, mouse monoclonal, Clontech 632380)
anti-G6PDH antibodies (1:50000, Sigma A9521)

Validation

All antibodies were tested in both infected and non-infected samples and are validated by the supplier as specific for the protein of interest.

Eukaryotic cell lines

Policy information about cell lines and Sex and Gender in Research

Cell line source(s)

A549 (ATCC CCL-185)
NuLi-1 (ATCC CRL-4011)
A549 mCherryER was generated in this work as describe in detail in the methods section.
HEK293E were kindly provided by Avinash R. Shenoy (Imperial College London)

Authentication

Cell lines were not authenticated.

Mycoplasma contamination

Cells routinely tested negative for Mycoplasma contamination.

Commonly misidentified lines
(See ICLAC register)

No commonly misidentified cell lines were used in the study.

Flow Cytometry

Plots

Confirm that:

- [x] The axis labels state the marker and fluorochrome used (e.g. CD4-FITC).
- [x] The axis scales are clearly visible. Include numbers along axes only for bottom left plot of group (a 'group' is an analysis of identical markers).
- [x] All plots are contour plots with outliers or pseudocolor plots.
- [x] A numerical value for number of cells or percentage (with statistics) is provided.

Methodology

Sample preparation	Yeast cells were prepared in PBS
Instrument	419 FACScan (Becton Dickinson) flow cytometer through a 488 nm excitation laser and a 585/42 BP emission filter (FL2)
Software	Data was processed using FlowJo software version V10CL (FlowJo LLC, Ashland, OR, USA)
Cell population abundance	Only yeast cells were present
Gating strategy	No need to gate the yeast cells

- [ ] Tick this box to confirm that a figure exemplifying the gating strategy is provided in the Supplementary Information.
